# Nanocomposite-Structured Sensing Interfaces on Fibrous Substrates for Chemiresistive Detection of VOCs

**DOI:** 10.3390/s26175369

**Published:** 2026-08-25

**Authors:** Lidia Gebre, Guojun Shang, Zeqi Li, Dong Dinh, Seyed Danial Mousavi, Madelyn Lee, Ielyzaveta Antonova, Jin Luo, Susan Lu, Cate Wisdom, Emily Long, Zakiya Skeete, Tony Yuan, Chuan-Jian Zhong

**Affiliations:** 1 Department of Chemistry, State University of New York at Binghamton, Binghamton, NY 13902, USA; lgebre1@binghamton.edu (L.G.); gshang1@binghamton.edu (G.S.); zli267@binghamton.edu (Z.L.); smousav2@binghamton.edu (S.D.M.); mlee231@binghamton.edu (M.L.); iantono1@binghamton.edu (I.A.); jluo@binghamton.edu (J.L.); zakiyaskeete@gmail.com (Z.S.); 2 Department of Systems Science and Industrial Engineering, State University of New York at Binghamton, Binghamton, NY 13902, USA; ddinh2@binghamton.edu (D.D.); slu@binghamton.edu (S.L.); 3 4D Bio^3^ Center for Biotechnology, Uniformed Services University, Bethesda, MD 20814, USA; ewisdom@genevausa.org (C.W.); elong@genevausa.org (E.L.); 4 The Geneva Foundation, Tacoma, WA 98402, USA; 5 Department of Radiology and Bioengineering, Uniformed Services University, Bethesda, MD 20814, USA

**Keywords:** chemiresistive sensors, nanocomposites, graphene, cellulose, gold nanoparticles, volatile organic compounds (VOCs), paper-based sensors, sensor arrays, chemiresistance, environmental monitoring

## Abstract

**Highlights:**

**What are the main findings?**

- Developed composition-programmable binary and ternary nanocomposite sensing interfaces on fibrous substrates that achieve high sensitivity and sub-ppm detection of volatile organic compounds (VOCs).
- Established interfacial polarity design rules where hydrophilic GE/CMC and amphiphilic GE/HEC interfaces enhance polar VOC responses, whereas hydrophobic gold thiolate assemblies target nonpolar analytes.

**What are the implications of the main findings?**

- Demonstrates a versatile strategy to modulate VOC affinity and sensor stability through physical integration, preserving cellulose matrix integrity while governing charge-transport pathways.
- Provides actionable guidelines for creating low-cost, scalable, and reduced-complexity paper-based chemiresistive sensor arrays ideal for portable environmental and occupational monitoring.

**Abstract:**

Nanocomposite-structured sensing interfaces were developed on fibrous substrates for chemiresistive detection of volatile organic compounds (VOCs) by integrating graphene (GE), cellulose derivatives hydroxyethylcellulose (HEC) and carboxymethylcellulose (CMC) and molecularly linked gold nanoparticles into composition-programmable thin films. Raman and infrared spectroscopy confirm that graphene incorporation occurs through physical integration without chemical modification of the polymer matrix, preserving cellulose integrity while enabling graphene loading to govern electrical percolation and charge-transport pathways. Systematic variation in nanocomposite composition reveals clear design rules linking interfacial polarity to VOC class sensitivity: hydrophilic GE/CMC and amphiphilic GE/HEC interfaces exhibit enhanced responses to polar and hydrogen-bonding VOCs, whereas hydrophobic gold thiolate assemblies preferentially respond to nonpolar aromatic and aliphatic VOCs. Incorporation of ligand-functionalized gold nanoparticles introduces an additional tunability dimension, modulating both VOC affinity and sensor stability through combined electronic and surface-chemical effects. Sensor arrays constructed from complementary nanocomposite interfaces achieve reliable VOC discrimination, as demonstrated by sensitivity patterns, spider-chart analysis, and principal component analysis, with effective separations retained even in reduced-sensor configurations. Across multiple nanocomposite architectures, enhanced response sensitivity is observed at low VOC concentrations, highlighting the role of interfacial adsorption dynamics and underscoring the potential of paper-based nanocomposite chemiresistive platforms for sub-ppm VOC detection.

## 1. Introduction

Air pollution is a major environmental contributor to declining human health, driven largely by anthropogenic emissions associated with fossil fuel production, waste burning, agricultural chemicals, automobile exhaust, tobacco smoke, and the widespread use of consumer solvents such as adhesives, paint thinners, and disinfectants [1,2,3,4,5]. A particularly concerning source arises from burn pits, which release harmful airborne vapors through the combustion of mixed waste streams including plastics and medical materials, generating toxic compounds such as dioxins, polycyclic aromatic hydrocarbons (PAHs), and volatile organic compounds (VOCs) [6,7]. Exposure to these VOCs and PAHs has been linked not only to respiratory disorders but also to long-term chronic health effects, especially among populations with repeated or prolonged exposure. Many VOCs emitted from these processes, including alkenes, aromatic hydrocarbons, and alcohols, are chemically persistent, readily transported over long distances, and include highly toxic or carcinogenic species. Air pollution, including combined ambient and household exposure, is associated with approximately 6.7 million premature deaths annually [8].

Current approaches for monitoring VOCs rely primarily on metal oxide semiconductor (MOS) sensors and laboratory-based analytical techniques. MOS sensors, which detect conductivity changes upon gas adsorption, have demonstrated utility for detecting a range of hydrocarbons from alcohols to ketones; however, limited selectivity, particularly for complex VOC mixtures, remains a persistent challenge [9,10,11]. Gas chromatography-mass spectrometry (GCMS) provides excellent chemical specificity but is constrained by high cost, maintenance requirements, the need for calibration standards, and poor portability, limiting its suitability for widespread or field-deployable monitoring [12,13]. These limitations underscore the need for low-cost, portable sensing platforms capable of selective and sensitive detection of anthropogenic VOCs. Chemiresistive sensors have emerged as a promising alternative owing to their simple device architecture, low fabrication cost, and potential for high sensitivity and rapid response. Early work demonstrated that chemiresistive sensing interfaces constructed from molecularly linked gold nanoparticles, prepared via exchange-crosslinking-precipitation reactions, could achieve high sensitivity and selectivity toward VOCs through controlled interparticle spacing and surface chemistry [14,15,16,17,18,19]. More recently, nanocomposite-structured sensing interfaces have attracted increasing attention as an alternative strategy [20,21,22,23]. By integrating diverse 1D, 2D, and 3D materials, such as graphene, polymers, and nanoparticles, nanocomposites offer expanded tunability of electrical transport, surface polarity, porosity, and chemical functionality, enabling selective interactions with different VOC classes.

Despite significant progress in chemiresistive sensing materials, a persistent challenge remains in establishing clear relationships between nanocomposite composition, interfacial structure, and VOC response characteristics, particularly in the context of sensor arrays designed for discrimination among chemically diverse analytes. In this work, we report the design and fabrication of binary and ternary nanocomposite-structured sensing interfaces on fibrous substrates for chemiresistive sensor arrays. The nanocomposite architectures integrate graphene, cellulose derivatives, and molecularly linked gold nanoparticles, and are evaluated using portable and wireless-compatible sensor platforms against a range of VOCs spanning hydrophilic and hydrophobic classes. While there have been studies of graphene-, cellulose-, and Au nanoparticle-based sensing materials, a knowledge gap exists regarding composition–structure–response relationships in nanocomposite sensing interfaces. Although functionalized graphene can exhibit modified electrical and chemical properties, surface functionalization also alters defect density, surface chemistry, and interactions with the polymer matrix, making it more difficult to isolate the individual contributions of nanocomposite composition to sensing behavior. The hypothesis of the present study is that clear design principles relating nanocomposite polarity and interfacial chemistry to VOC sensing characteristics can be established by employing graphene within composition-programmable nanocomposite architectures. The graphene serves as the conductive framework of the nanocomposite, whereas the cellulose derivatives (CMC and HEC) and ligand-functionalized Au nanoparticles are responsible for tailoring the interfacial polarity, adsorption characteristics, and overall sensing selectivity.

Key findings include (i) composition-programmable nanocomposite sensing interfaces on fibrous substrates by integrating graphene, cellulose derivatives (CMC, HEC), and molecularly linked gold nanoparticles, enabling systematic tuning of electrical percolation, surface polarity, and interfacial interactions relevant to VOC detection; (ii) physical nature of graphene–cellulose interactions, as confirmed by Raman and infrared spectroscopy, allowing graphene loading to govern charge-transport pathways and chemiresistive sensitivity; (iii) distinct design rules linking nanocomposite’s VOC sensitivity to hydrophilic GE/CMC and amphiphilic GE/HEC interfaces for enhanced responses to polar and hydrogen-bonding VOCs, whereas hydrophobic Au-thiolate assemblies for preferential responses to nonpolar aromatic and aliphatic VOCs; (iv) incorporation of ligand-functionalized Au NPs for additional tunability dimension, modulating both VOC affinity and sensor stability through combined electronic and surface-chemical effects; (v) nanocomposite array interfaces to achieve VOC discrimination, demonstrating effective separations; and (vi) enhanced response sensitivity at low VOC concentrations across multiple nanocomposite architectures, providing a pathway toward sub-ppm detection using paper-based chemiresistive platforms. The primary objective of the present work is to establish composition–structure–response relationships for composition-programmable nanocomposite sensing interfaces rather than to optimize a particular sensing material for the fastest response. Consequently, the work focuses on systematic comparisons of sensitivity, concentration-dependent response, and array-level discrimination across different nanocomposite architectures.

## 2. Materials and Methods

### 2.1. Chemicals

1,9-nonanedithiol (NDT, 95%), decanethiol (DT, 96%), hydrogen tetracholoroaurate (HAuCl_4_, 99%), tetraoctylammonium bromide (TOABr, 99%), sodium borohydride (NaBH_4_, 99%), sodium carboxymethylcellulose (CMC), hydroxyethylcellulose (HEC), and graphene nanoplatelets (GE). Vapors were generated from hexane (Hx, 99.9%), benzene (Bz, 99.0%), toluene (Tl, 99.9%), ethanol (EtOH, 100%). The water was purified with a Millipore Milli-Q water system.

### 2.2. Preparation of Chemiresistive Arrays

Interdigitated microelectrodes (IMEs) were fabricated on photopaper substrates by screen printing of silver or silver-copper nanopastes using screen-printing method (a stainless steel 325-micron mesh) [24] and inkjet printing of silver nanoink using Dimatix printer. The microelectrode parameters for the nanocomposite-structured sensors feature microelectrode spacing, width, and length of 136, 184, and 3050 µm, respectively. The nanostructured-assembled sensors had a spacing, width, and length of 160, 240, and 2200 µm, respectively.

The nanocomposite structures and compositions are illustrated in Table S1. In Array-1, three of the sensors were prepared via the exchange-crosslinking-precipitation method [20]. Crosslinking was performed using NDT and 2 nm gold nanoparticles synthesized via a two-phase method. Inkjet-printed IMEs on photo paper were used as a substrate. The substrates were submerged in solution for approximately 45 h. The other five sensors were prepared via spray method onto screen-printed IMEs, with three of them containing GE and CMC and two of them containing GE and HEC. Each of these sensors was sprayed at different times, which led to thin films with variable resistances (see Figure S1A). A representative image of the screen-printed IME Array-2 was designed to investigate changes in the sensitivity and selectivity between binary and ternary nanocomposite sensing interfaces. Four sensors were binary composites using GE, HEC, and CMC using the same method as sensors 1-3, 4-5 in array 1. The other 3 sensors were prepared with these components in different combinations, then spiked with gold nanoparticles capped with different linker molecules. All components of the second array were prepared via spray method.

Descriptions of the Arrays (Table S1): The order of the sensors was based on the film type, not the sensor position number in the array socket.

In the 8-sensor array socket (Array-1), the sensors are arranged linearly in a row from 1 to 8 and designated as S-1 through S-8. These sensors are classified into three distinct categories based on their chemical composition and structural type:

i.   Group 1 (GE/CMC): Comprises sensors S-1, S-3, and S-2, subdivided into groups 1a, 1b, and 1c, respectively.
ii.  Group 2 (GE/HEC): Comprises sensors S-5 and S-8, subdivided into groups 2a and 2b, respectively.
iii. Group 3 (Au2nm-NDT): Comprises sensors S-4, S-7, and S-6, subdivided into groups 3a, 3b, and 3c, respectively.

In the second array configuration (Array-2), the socket accommodates 7 functional sensors labeled from S-1 to S-8 (with S-5 left unassigned). These sensors are classified into five distinct categories based on their chemical composition and structural type:

i.   Group 1 (GE/CMC): Comprises sensors S-2 and S-1, subdivided into groups 1a and 1b, respectively.
ii.  Group 2 (GE/HEC): Comprises sensors S-3 and S-4, subdivided into groups 2a and 2b, respectively.
iii. Unassigned (S-5): The position for Sensor S-5 contains no sensor.
iv. Group 4 (Au2nm-DT / GE/CMC): Contains sensor S-8, designated as group 4a.
v.   Group 5 (Au2nm-DT / GE/HEC): Contains sensor S-6, designated as group 5a.
vi.  Group 6 (Au2nm-DT / MUA / GE/HEC): Contains sensor S-7, designated as group 6a.

### 2.3. Experimental Measurements

The goal of the sensitivity tests was to understand how varying components of the film affected the sensor’s overall sensitivity to gases ranging from hydrophobic, amphiphilic, and hydrophilic. The sensor arrays were tested using the following VOCs: toluene, benzene, ethanol, and hexane, as well as water. Array 1 was exposed to each vapor at 5 different concentrations in increasing order. Array 2 was exposed to 4 of these vapors, excluding water. Each exposure was performed in triplicate. N_2_ gas (99.99%, Airgas) was used to dilute the vapor and to decontaminate the system after each exposure. Calibrated mass-flow controllers (AFC-2600, Aalborg Instruments & Controls, Inc., Orangeburg, NY, USA) were used for the vapor generation. The vapor concentration (ppm, moles per liter) was calculated from the partial vapor pressure and the mixing ratio of vapor and N_2_ flows. Two gas-mixing and flow conditions were employed, including a 2-channel dilution to achieve a total flow rate of 200 mL/min for vapor concentrations above a few hundred and up to a few thousand ppm, and a 3-channel mixing/dilution to achieve a total flow rate of 400, 800, and 1600 mL/min for vapor concentrations down to low ppm. Before the start of each test, the array was purged for 55 min to establish a baseline.

A wireless and low-current sensor electronic prototype developed in our laboratory [14,15] was used for the measurement of the sensor responses. The sensor array was interfaced with a sensor electronic board coupled with a wireless circuit board for data acquisition, which was further processed by software for data processing.

In a typical sensor array, 4–10 sensors with their electrical leads aligned on one end of the paper chip for inserting into the connecting port of the sample manifold, which is then connected to our integrated electronic detection circuit board. The sensor array was interfaced with a benchtop device or our electronic board coupled with a wireless circuit board for data acquisition. The data were processed by our pattern recognition algorithm/software. Spider chart analysis and PCA analysis methods were used to enhance the identifications. The raw data were extracted from the cloud and pre-processed by data pre-processing techniques prior to processing and analysis.

## 3. Results and Discussion

### 3.1. Characterization of Nanocomposite Sensing Films on Fibrous Substrates

The structural and chemical characteristics of the nanocomposite sensing films were systematically examined to establish the composition, integrity, and phase distribution of the graphene–cellulose interfaces used for sensor array construction. A combination of Raman spectroscopy and infrared spectroscopy was employed to confirm successful incorporation of graphene within the cellulose-based matrix, evaluate composition-dependent structural evolution, and assess whether graphene integration induces chemical modification of the polymer host. These analyses provide a foundation for interpreting the chemiresistive sensing behavior.

#### 3.1.1. Nanocomposite Fabrication and Morphology

**Figure 1:**
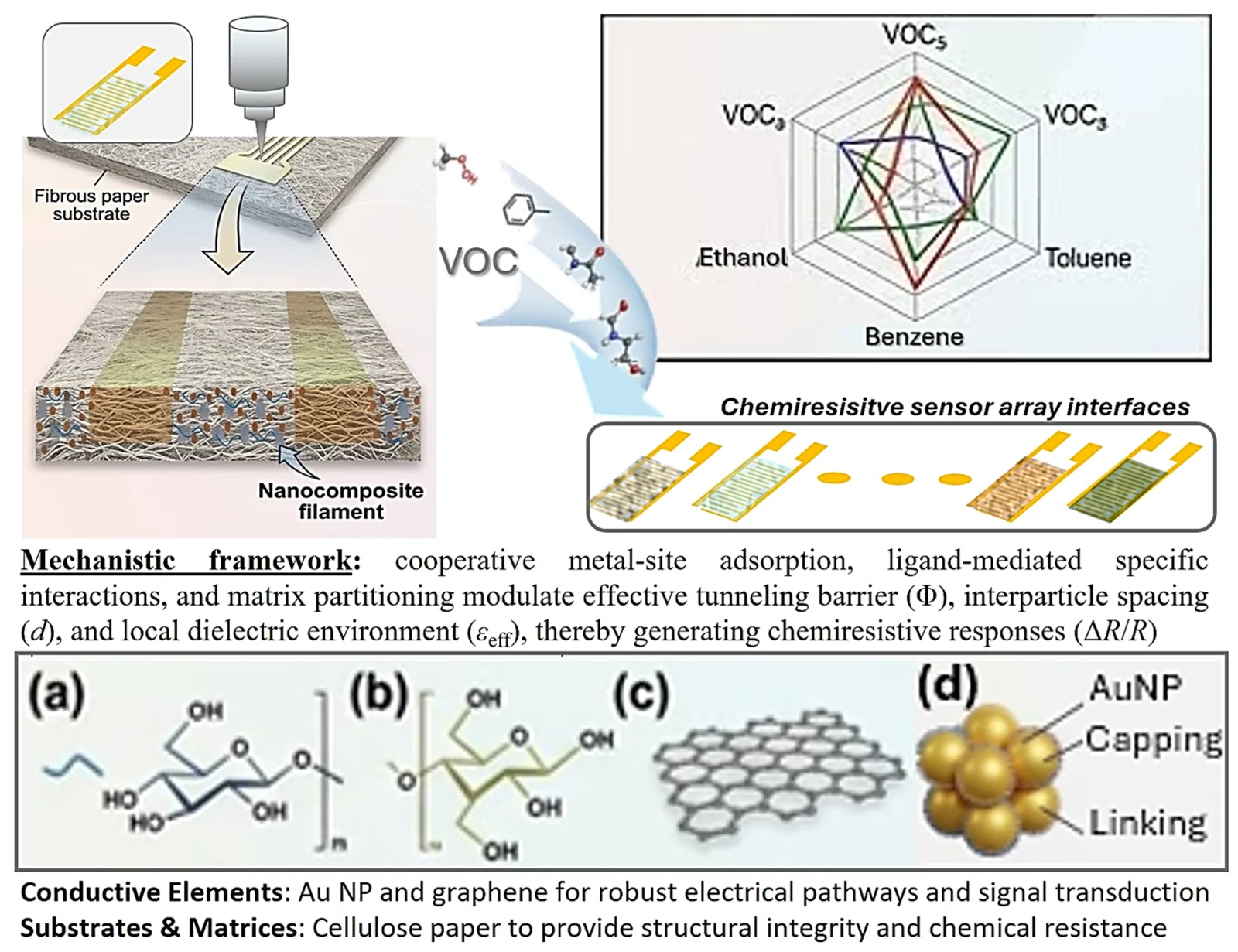
Schematic illustration of the interfacial structures of the nanocomposite sensing films for detection of VOCs through array chemiresistive transduction mechanism. Nanocomposite components: (**a**) sodium carboxymethylcellulose (CMC), (**b**) hydroxyethylcellulose (HEC), (**c**) graphene nanoplatelets (GE), and (**d**) molecularly linked gold nanoparticles capped with different capping and linking molecules (e.g., DT-Au_nm_, NDT-Au_nm_, MUA-Au_nm_). The composition-programmable nanocomposite sensing interfaces are designed on fibrous substrates by integrating graphene, cellulose derivatives (CMC or HEC), and molecularly linked gold nanoparticles, enabling systematic tuning of electrical properties, surface polarity, and interfacial interactions for VOC detection.

The nanocomposite sensing films are constructed from nanocomposite ink formulations by mixing CMC (or HEC), GE, and molecularly linked or capped gold nanoparticles of different sizes (e.g., Au_2nm_, Au_5nm_) capped with alkyl thiols (e.g., DT) and linked with functional alkyl thiols (e.g., NDT, MUA) with controlled concentrations and ratios. The ink was then deposited as thin films on the interdigitated microelectrode device by controlled spraying. Figure 1 shows the structures of these components for formulating the nanocomposite inks and fabricating the chemiresistive nanocomposite thin films on interdigitated microelectrode array devices.

The nanocomposites offer the flexibility of the sensor arrays in terms of the various structural properties, including hydrophobicity/hydrophilicity, polar/non-polar, aromatic/aliphatic, porous/non-porous, electrostatic-bonding, hydrogen-bonding, ligand coordination, for interactions of the nanocomposite film with the VOC analytes. The thermally activated semiconductive electrical properties of the nanocomposite films are tuned by the various combinations of the conductive graphene (GE) [25,26], non-conductive cellulose (CMC or HEC) [27,28], and semiconductive nanoparticle assemblies. Various combinations were explored for the design of sensor arrays, including (1) conductive GE in highly hydrophilic cellulose structure (e.g., GE/CMC), (2) conductive GE in hydrophobic-hydrophilic cellulose structure (e.g., GE/HEC), (3) hydrophobic assemblies of Au nanoparticles (e.g., NDT-Au2nm, MUA-Au2nm), (4) hydrophobic Au nanoparticles in GE/CMC (e.g., DT-Au2nm/GE/CMC, NDT-Au2nm/GE/CMC), (5) hydrophobic Au nanoparticles in GE/HEC (e.g., DT-Au2nm/GE/HEC, NDT-Au2nm/GE/HEC), and (6) hydrophobic/hydrophilic Au nanoparticles in GE/HEC (e.g., MUA-Au2nm/GE/HEC) or in GE/CMC (e.g., MUA-Au2nm/GE/CMC).

#### 3.1.2. Film Thickness, Penetration, and Connectivity

The overall morphologies of the nanocomposite thin films were first examined using AFM and SEM microscopic techniques to characterize the 3D nanofilament structure and inter-filament connectivity in the fibrous structure. While the overall penetration of nanoinks depends strongly on the porosity of the fibrous substrates, there are subtle differences in nanoink-coating structure interactions, which are confirmed by SEM images and EDX analysis of a nanoink-printed IME device on a low-porosity photopaper substrate. A comparison of the cross-thickness image contrast and the elemental line profiles clearly demonstrates that the layer directly under the conductive silver layer differs from that with the silver layer. We investigated various methods to prepare the sensor arrays. The spraying-prepared film thickness was analyzed by AFM (Figure S1B,C). Thicknesses of the thin films were controllable in the range from 100 nm to 3000 nm, as determined by cross-section analysis of the AFM images of the sensor microelectrodes.

We also characterized the electrical properties of the nanoink-printed fibrous devices and the nanofilament penetration depths and distribution in the fibrous structures of different fibrous substrates. The measured electrical resistance depends on the amount of MUA-Au_2nm_ component in the nanocomposite ink with CMC and GE in controlled ratio (Figure S2), showing controllability of the thin film resistance by the composition of the nanocomposite. This demonstrates that the composition-programmable nanocomposite stems from the electrical tunability by graphene, cellulose derivatives (CMC or HEC), and molecularly linked gold nanoparticles.

#### 3.1.3. Raman and FTIR Confirmation of Composite Formation

**Figure 2:**
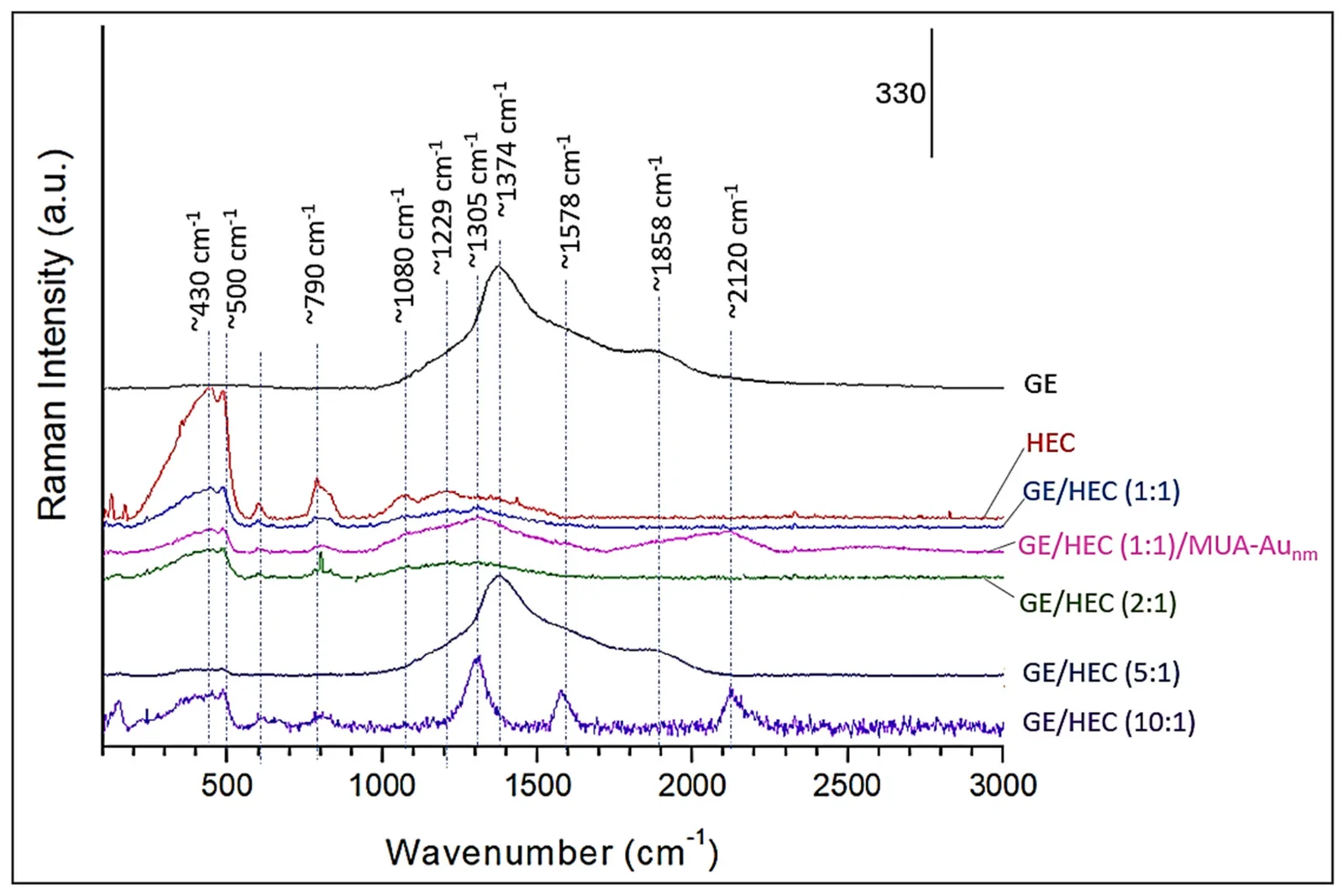
Raman spectra of pure HEC, commercial graphene (GE), and GE/HEC nanocomposite films with varying GE-to-HEC ratios deposited on gold substrates, highlighting the evolution of graphene-related features with increasing GE-to-HEC molar ratios and the incorporation of MUA-linked assemblies of Au NPs capped with DT (MUA-Au_nm_). Note that the insertion of the GE/HEC/MUA-Au_nm_ curve into the GE/HEC ratio series is to highlight the contrast of the MUA-Au-modified 1:1 sample with the GE/HEC ratio series, to more clearly illustrate the composition-dependent evolution while distinguishing the Au-modified sample from the GE:HEC ratio series. The progressive attenuation of cellulose-associated vibrational modes and the emergence of graphene D and G bands confirm successful incorporation of graphene and composition-dependent transition from polymer-dominated to graphene-dominated scattering behavior. Their corresponding vibrational assignments (e.g., O–H stretching, C–H stretching, C=O stretching, COO^−^ asymmetric stretching, C–O–C stretching) are described in the accompanying discussion.

Raman spectroscopy was employed to confirm the formation of the nanocomposite structure and to examine the relative contributions of the polymer matrix and graphene filler. Representative spectra of pure HEC, commercial graphene (GE), and GE/HEC composite films deposited on gold substrates are shown in Figure 2. The Raman spectrum of HEC is dominated by characteristic cellulose vibrational modes in the 300–1800 cm^−1^ region, including a strong band at ~430 cm^−1^ attributed to skeletal bending of the pyranose ring, weaker features near ~600 cm^−1^ associated with ring and C–O bending, and a medium-intensity band at ~820 cm^−1^ corresponding to C–C/C–O stretching and CH_2_ rocking modes. Broad, overlapping features between ~1050 and 1600 cm^−1^ arise from coincident glycosidic C–O–C stretching, C–H bending, and O–H deformation modes typical of modified cellulose [29,30]. In contrast, the Raman spectrum of commercial GE exhibits broad, overlapping carbon bands between ~1200 and 2100 cm^−1^, with a dominant D band centered near ~1370 cm^−1^ and a weaker G band near ~1580 cm^−1^. The strong D-band intensity reflects a high defect density and edge-rich structure, consistent with chemically processed or multilayer commercial graphene.

The evolution of Raman spectra with increasing GE:HEC ratio clearly reflects a transition from polymer-dominated to graphene-dominated scattering. At low GE contents (1:1 and 2:1 GE/HEC), characteristic HEC peaks remain visible but are progressively attenuated, while a broad carbon-related envelope emerges in the 1000–1600 cm^−1^ region due to overlap between graphene D/G bands and polymer vibrational modes [26]. At higher GE contents (5:1 and 10:1 GE/HEC), cellulose-related peaks in the low-wavenumber region (~420, ~600, and ~820 cm^−1^) largely disappear, but remain observable. The spectra become dominated by graphene features. In particular, the 10:1 GE/HEC film exhibits clearly resolved D (~1310 cm^−1^) and G (~1578 cm^−1^) bands, indicating that graphene is the primary Raman scattering phase [31,32]. The increasing prominence of graphene bands with GE loading is attributed to both the higher Raman scattering cross-section of sp^2^ carbon and optical shielding of the polymer matrix by graphene flakes. Spectral subtraction of HEC contributions further confirms the progressive emergence of graphene-dominated signatures with increasing GE content. These results collectively demonstrate successful incorporation of graphene within the cellulose matrix and the formation of composition-tunable nanocomposite sensing films.

**Figure 3:**
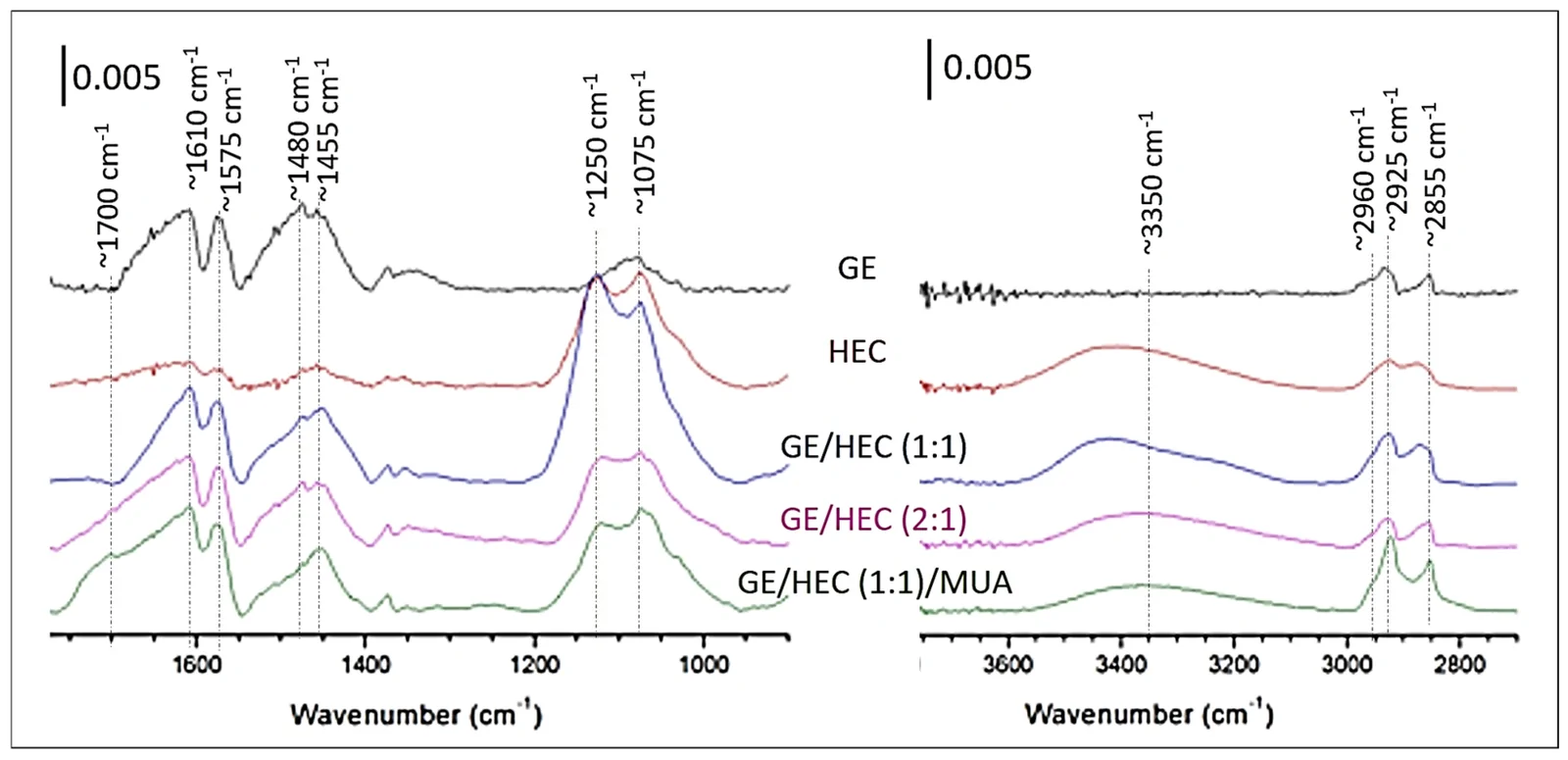
Infrared reflection-absorption spectra (IRRAS) of pure HEC, commercial graphene (GE), and GE/HEC nanocomposite films deposited on gold-coated fibrous substrates. Characteristic cellulose vibrational modes are retained in the composite films, while their relative intensities decrease with increasing GE content. The absence of new absorption bands or systematic peak shifts indicates that graphene incorporation occurs without chemical modification of the polymer matrix. See Figure S3 for GE/CMC films. Their corresponding vibrational assignments are described in the accompanying discussion.

Infrared spectroscopy (IRRAS) was used to further confirm the chemical composition of the nanocomposite sensing films and to assess the interaction between the cellulose-based matrix and graphene fillers. Figure 3 shows representative IRRAS spectra of pure HEC, GE, and GE/HEC composite films deposited on gold-coated fibrous substrates. The IRRAS spectrum of pure HEC exhibits characteristic absorption bands of hydroxyethyl-substituted cellulose, including a broad O–H stretching band centered near ~3400 cm^−1^, C–H stretching modes at ~2900 cm^−1^, and a series of strong bands between ~1000 and 1200 cm^−1^ corresponding to C–O–C and C–O stretching vibrations of the polysaccharide backbone. There are some very weak peaks near ~1450 cm^−1^ and ~1600 cm^−1^ due to cellulose derivatives [29,30]. The IR spectrum of commercial GE showed some skeletal vibrations of the carbon lattice and some residual functional groups associated with unoxidized sp^2^ C=C bonds within GE aromatic rings (1607 cm^−1^), aromatic C=C stretching from “G-band” equivalent IR band for crystalline structures (1575 cm^−1^), C-H bending or aromatic C=C stretching of the carbon network (1478 cm^−1^), C-O stretching likely from residual epoxy or alkoxy groups (1075 cm^−1^), and weak C–H stretching at ~2900 cm^−1^ due to residual defects in the GE. The weak and broad absorptions associated with residual oxygen-containing functional groups (e.g., C–O and O–H vibrations) are observed, consistent with partially oxidized or defect-containing commercial graphene [31,32]. In comparison, the IR spectra of GE/HEC composite films retain the major vibrational features of HEC, confirming that the polymer matrix remains chemically intact upon incorporation of graphene. As the GE fraction increases, the relative intensity of HEC-associated absorption bands, particularly the O–H stretching (~3400 cm^−1^) and C–O–C stretching (1000–1200 cm^−1^) modes, gradually decreases. This attenuation is attributed primarily to the increasing optical shielding effect of graphene flakes and the reduced effective volume fraction of HEC in the film, rather than to chemical degradation of HEC.

In contrast to GE/HEC films, the GE/HEC/Au-MUA film showed a clear shoulder peak at 1704 cm^−1^, which is characteristic of the CO_2_H stretching band from the terminal carboxylic acid groups in the Au-MUA assembly. This observation, along with the observation of the -CH_3_ asymmetric stretching band at ~1955 cm^−1^ associated with the capping DT in the MUA/DT mixed monolayer on the Au NPs, demonstrates the successful incorporation of MUA-Au in the GE/HEC network. The same C-H and O-H stretching was observed in CMC and 1:1 GE/CMC (Figure S3), where CMC (carboxymethylcellulose) features highly hydrophilic structures in comparison with HEC. The GE/CMC displayed peaks corresponding to the asymmetric and symmetric stretching of carboxylate groups (~1422 and ~1624 cm^−1^) associated with CMC [33,34] and peaks associated with GE (~1575 and 1478 cm^−1^). No new absorption bands or systematic peak shifts are observed upon graphene incorporation, indicating the absence of covalent bonding or chemical transformation between GE and the cellulose matrix. The interaction between GE and HEC is therefore dominated by physical mixing and noncovalent interactions, such as hydrogen bonding and van der Waals forces. These interactions are sufficient to stabilize a percolated graphene network within the fibrous polymer matrix while preserving the chemical identity of each component.

The IRRAS results complement Raman analysis by confirming successful formation of composition-tunable GE/HEC nanocomposite films and by establishing that graphene incorporation primarily alters the structural and electronic landscape of the sensing interface without inducing chemical modification of the polymer host. This structural integrity is critical for ensuring reproducible chemiresistive responses in subsequent VOC sensing studies. Both Raman and infrared characterizations demonstrate that the nanocomposite sensing films consist of physically integrated yet chemically distinct graphene and cellulose phases, with graphene content governing the degree of electrical percolation and interfacial connectivity. The absence of chemical modification to the polymer host ensures structural stability and reproducibility, while the tunable graphene loading provides a means to systematically modulate charge transport pathways and interparticle junctions. Importantly, the absence of new absorption bands or significant peak shifts upon graphene incorporation indicates that the graphene is physically integrated within the cellulose matrix rather than covalently bonded, consistent with the Raman analysis. These characteristics are directly relevant to the chemiresistive response of the sensor arrays and form the basis for the VOC sensing performance discussed in the next section.

### 3.2. Sensor Response Characteristics

#### 3.2.1. Polarity-Driven Response Trends in Nanocomposite Sensor Arrays

The chemiresistive response characteristics of Array 1 were systematically evaluated using VOCs spanning polar and hydrophilic (water, ethanol), hydrophobic (toluene), and nonpolar (benzene, hexane) chemical classes. For each vapor, the sensor response profiles and corresponding sensitivity plots were obtained over five concentration levels, with three repeated measurements to assess reproducibility. Linear regression of the relative resistance change (ΔR/R_i_) versus vapor concentration was used to quantify sensor sensitivity.

**Figure 4:**
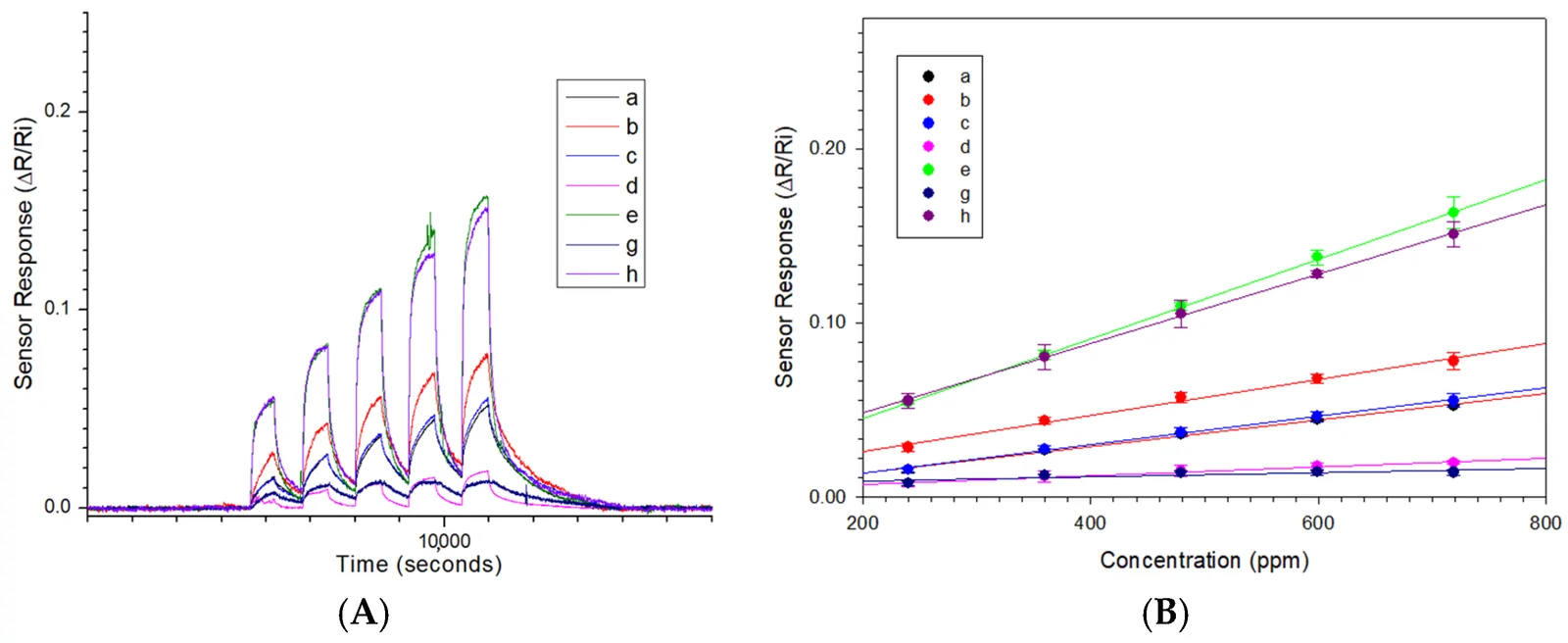

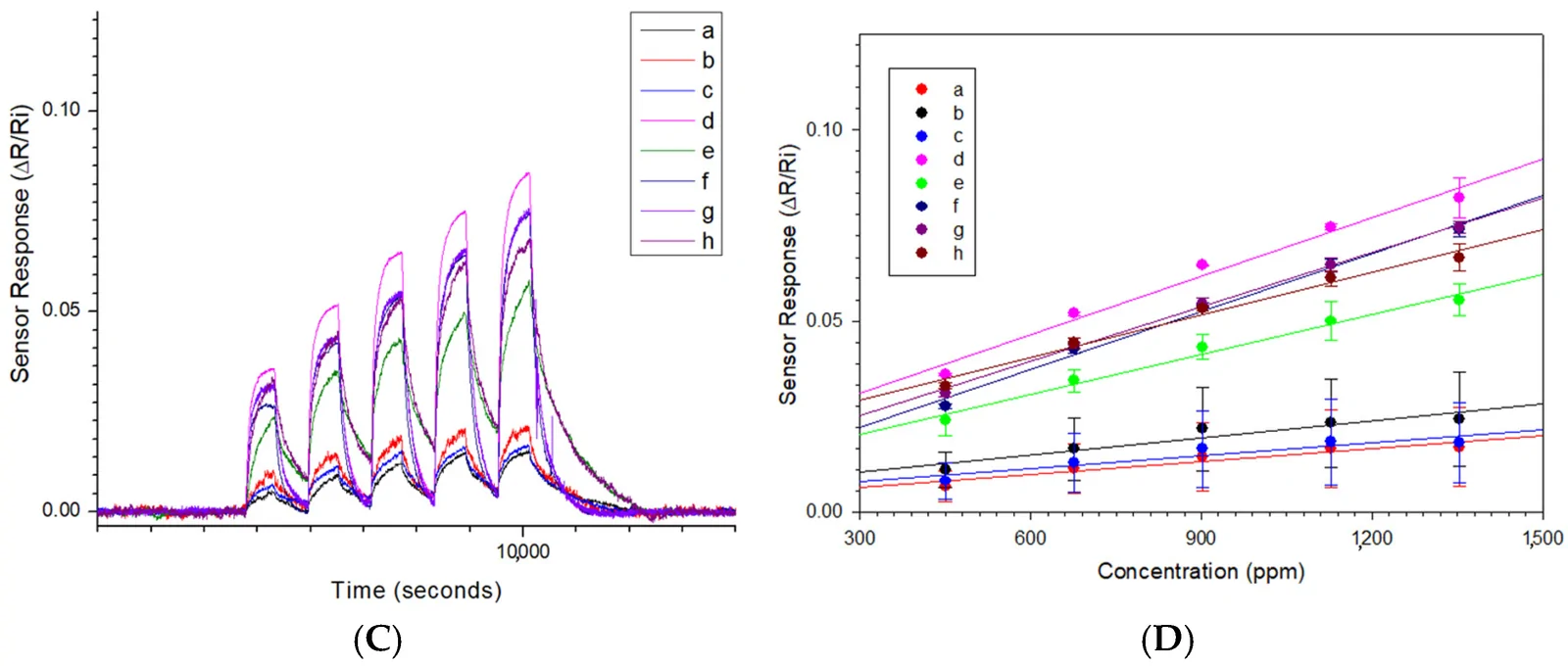
Sensor responses and sensitivities in response to ethanol (**A**,**B**) and benzene (**C**,**D**) vapors (array 1). (**A**,**B**): Data for three replica measurements are shown. (**A**) Sensor (S) response profile of a: S-1; b: S-2; c: S-3; d: S-4; e: S-5; g: S-7; h: S-8; and (**B**) sensitivity (1/ppm) plot against ethanol vapor (a: 7.21 × 10^−5^; b: 1.06 × 10^−4^; c: 8.46 × 10^−5^; d: 2.47 × 10^−5^; e: 2.30 × 10^−4^; g: 1.30 × 10^−5^; h: 2.01 × 10^−4^). Sensor S-6 had no clear response to ethanol and was excluded from the plot. (**C**,**D**): (**C**) sensor response profile of a: S-1; b: S-2; c: S-3; d: S-4; e: S-5; f: S-6 g: S-7; h: S-8; and (**D**) sensitivity (1/ppm) plot of array 1 against benzene vapors (a: 1.15 × 10^−5^; b:1.49 × 10^−5^; c: 1.13 × 10^−5^; d: 5.10 × 10^−5^; e: 3.47 × 10^−5^; f: 5.06 × 10^−5^; g: 4.75 × 10^−5^; h: 3.73 × 10^−5^). This dataset demonstrates distinct design rules derived from linking nanocomposite polarity to VOC class sensitivity: hydrophilic GE/CMC and amphiphilic GE/HEC interfaces exhibit enhanced responses to polar and hydrogen-bonding VOCs, whereas hydrophobic Au-thiolate assemblies preferentially respond to polar and nonpolar aromatic and aliphatic VOCs.

Clear polarity-dependent response trends emerge from the dataset (Figure 4 and Figure S4). For polar and hydrogen-bonding vapors such as ethanol and water, sensors incorporating graphene–cellulose nanocomposites (GE/CMC and GE/HEC) exhibit substantially higher response magnitudes and sensitivities compared to hydrophobic Au-NDT nanoparticle films. Among the graphene–cellulose interfaces, GE/HEC sensors consistently display higher sensitivity toward ethanol than GE/CMC sensors (Figure 4A,B), reflecting the amphiphilic nature of HEC, which enables favorable interactions with both the hydroxyl functionality and the alkyl backbone of ethanol molecules. In contrast, the more strongly hydrophilic GE/CMC interfaces show the highest sensitivity toward water vapor (Figure S4A,B), consistent with the presence of terminal carboxylate groups that enhance hydrogen-bonding and dipole–dipole interactions.

For nonpolar and weakly polar vapors such as benzene, toluene, and hexane, the response trends reverse (Figure 4C,D and Figure S4C–F). Sensors based on hydrophobic Au-NDT assemblies exhibit the highest sensitivities, followed by GE/HEC and GE/CMC sensors. The enhanced response of Au-NDT films toward aromatic and aliphatic hydrocarbons is attributed to favorable van der Waals interactions between nonpolar VOCs and the alkyl thiolate ligands capping the gold nanoparticles. In contrast, GE/CMC sensors often display reduced sensitivity and, in some cases, gradual response decay during prolonged exposure to hydrophobic vapors, indicative of short-term percolation or structural reorganization within the hydrophilic nanocomposite network.

These results establish a clear and reproducible design rule linking nanocomposite interfacial polarity to VOC class sensitivity: hydrophilic and amphiphilic graphene–cellulose interfaces preferentially enhance responses to polar and hydrogen-bonding VOCs, whereas hydrophobic nanoparticle assemblies preferentially respond to nonpolar aromatic and aliphatic vapors.

#### 3.2.2. Role of Graphene–Cellulose Interfaces and Nanocomposite Architecture

Beyond polarity matching, the response characteristics of the graphene–cellulose nanocomposites are strongly influenced by the structural role of graphene within the polymer matrix. In both GE/CMC and GE/HEC sensors, graphene provides electrically conductive pathways that enable chemiresistive transduction, while the cellulose derivatives define the chemical environment governing VOC adsorption. Variations in film thickness and graphene loading result in measurable differences in baseline resistance, response magnitude, and stability, consistent with modulation of electrical percolation pathways within the nanocomposite network.

Comparative analysis of GE/CMC and GE/HEC sensors highlights the importance of polymer chemistry in defining interfacial interactions. Although both matrices support graphene dispersion and electrical connectivity, GE/HEC sensors generally exhibit higher sensitivity toward hydrophilic VOCs and improved response stability across repeated exposure cycles. This behavior is attributed to the hydroxyethyl substituents of HEC, which introduce amphiphilic character and reduce excessive swelling or restructuring during vapor adsorption. In contrast, GE/CMC sensors, while highly sensitive to strongly polar vapors such as water, are more prone to response decay when exposed to hydrophobic VOCs, reflecting transient disruption of conductive pathways due to unfavorable interfacial interactions.

An assessment based on Langmuir-type adsorption provides additional insight into these trends. Fits of selected response profiles to a Langmuir adsorption model (Figure S10) reveal that sensors exhibiting higher response sensitivities also display faster apparent adsorption kinetics, indicating that adsorption dynamics at the nanocomposite–VOC interface play a critical role in governing chemiresistive performance. While the simplified model does not capture all aspects of nanocomposite restructuring, it supports the interpretation that both chemical affinity and interfacial transport kinetics contribute to the observed sensitivity trends.

These findings demonstrate that graphene–cellulose nanocomposites function as chemically programmable sensing interfaces in which the polymer matrix controls VOC affinity while graphene governs electrical percolation and signal transduction. This synergistic interplay between composition, polarity, and network connectivity provides a versatile platform for tailoring chemiresistive responses across diverse VOC classes.

#### 3.2.3. Tunability and Stability Modulation by Ligand-Functionalized Nanoparticles

**Figure 5:**
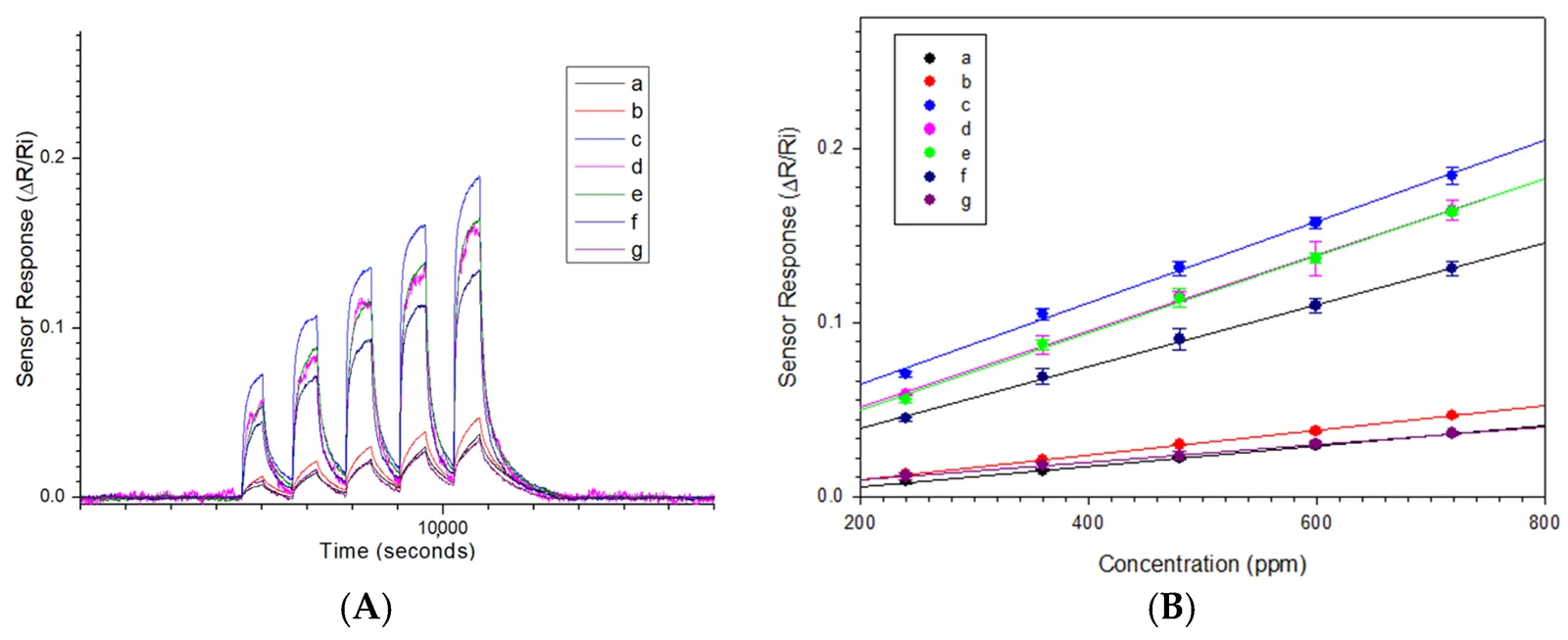

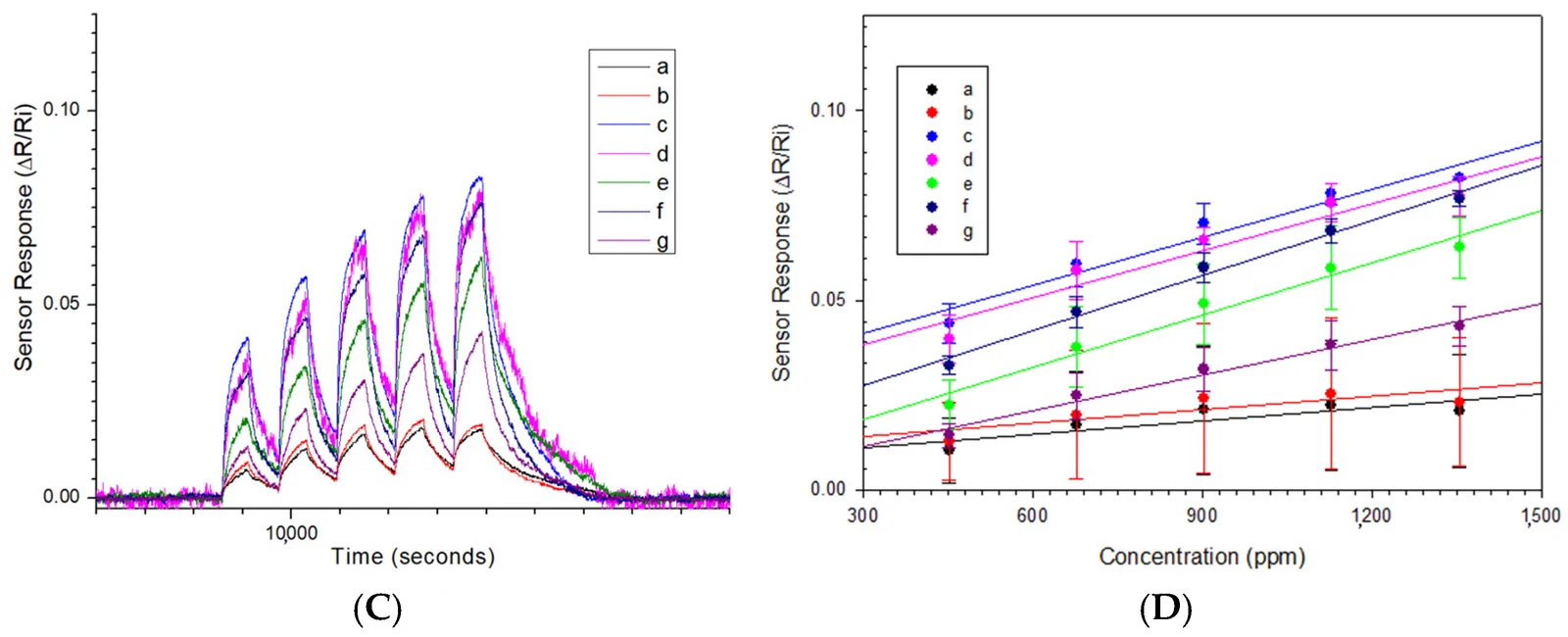
Sensor responses and sensitivities in response to ethanol (**A**,**B**) and benzene (**C**,**D**) vapors (array 2). (**A**,**B**): (**A**) Sensor (S) response profile of a: S-1; b: S-2; c: S-3; d: S-4; e: S-6; f: S-7; g: S-8; and (**B**) sensitivity (1/ppm) plot against ethanol vapor (a: 5.97 × 10^−5^; b: 7.02 × 10^−5^; c: 2.40 × 10^−4^; d: 2.27 × 10^−4^; e: 2.24 × 10^−4^; f: 1.81 × 10^−4^; g: 5.12 × 10^−5^). (**C**,**D**): (**C**) Sensor response profile of array 2: a: S-1; b: S-2; c: S-3; d: S-4; e: S-6; f: S-7; g: S-8; and (**D**) sensitivity plot (1/ppm) against benzene vapors (a: 1.35 × 10^−5^; b: 1.36 × 10^−5^; c: 3.64 × 10^−5^; d: 4.03 × 10^−5^; e: 4.67 × 10^−5^; f: 4.84 × 10^−5^; g: 3.26 × 10^−5^). This dataset demonstrates an additional tunability dimension for modulating VOC affinity through combined electronic and surface-chemical effects by incorporation of ligand-functionalized gold nanoparticles.

To further expand the tunability of nanocomposite sensing interfaces, ligand-functionalized gold nanoparticles were incorporated into graphene–cellulose matrices to form ternary nanocomposites. Array 2 was designed to evaluate how Au nanoparticle incorporation modifies VOC affinity, response sensitivity, and signal stability through combined electronic and surface-chemical effects. Sensor responses and sensitivities were examined for ethanol and benzene vapors, with representative data shown in Figure 5 and additional results provided in Figures S5 and S6.

For ethanol vapor, sensors incorporating GE/HEC matrices exhibit higher response sensitivities than their GE/CMC counterparts, consistent with trends observed in Array 1 (Figure 5A,B). Introduction of Au nanoparticles into GE/HEC films leads to modest but systematic changes in sensitivity, with Au-DT-modified sensors showing slightly reduced response magnitudes relative to unmodified GE/HEC, and Au-MUA-modified sensors exhibiting further attenuation. These trends reflect the competing influences of polymer-VOC interactions and nanoparticle surface chemistry, where incorporation of hydrophobic or weakly hydrophilic ligands partially suppresses hydrogen-bonding interactions between ethanol and the cellulose matrix. In contrast, incorporation of Au nanoparticles into GE/CMC films produces comparatively smaller changes in ethanol sensitivity, indicating that the strongly hydrophilic CMC environment dominates adsorption behavior in these composites.

For benzene vapor, the effect of Au nanoparticle incorporation is more pronounced (Figure 5C,D). Sensors containing GE/HEC films spiked with ligand-functionalized Au nanoparticles display enhanced response stability and improved linearity across repeated exposure cycles relative to unmodified GE/HEC sensors. Notably, GE/HEC sensors incorporating Au-MUA maintain linear response behavior throughout all cycles, whereas several sensors without Au nanoparticles exhibit loss of linearity or response decay at higher benzene concentrations. This stabilization effect is attributed to additional interfacial interactions introduced by the Au nanoparticles, which can reinforce network connectivity and mitigate transient percolation or restructuring during adsorption of hydrophobic VOCs.

The influence of Au nanoparticle incorporation extends beyond sensitivity modulation to include improved signal robustness during exposure to hydrophobic vapors such as toluene and hexane (Figure S5). In these cases, Au-modified GE/HEC sensors consistently exhibit reduced response decay and faster recovery compared to graphene–cellulose composites without Au nanoparticles. The presence of ligand-functionalized Au nanoparticles is therefore inferred to stabilize conductive pathways and reduce susceptibility to short-term structural rearrangements induced by weakly interacting VOCs.

These results demonstrate that ligand-functionalized gold nanoparticles introduce an additional and independent design parameter for tuning nanocomposite sensing interfaces. By appropriate selection of ligand chemistry and nanoparticle incorporation, it is possible to modulate VOC affinity, sensitivity, and response stability without fundamentally altering the underlying graphene–cellulose architecture. This tunability complements the polarity-driven design rules established in the earlier sections and enables rational optimization of chemiresistive sensor arrays for chemically diverse VOC environments.

In addition to modulating VOC affinity and sensitivity, incorporation of ligand-functionalized Au nanoparticles enhances response stability and reproducibility. Repeated exposure and mild annealing during sensing cycles reduce response noise and suppress transient percolation effects within the nanocomposite network, leading to improved linearity and cycle-to-cycle consistency (Figure S6). These stabilization effects further support the role of Au nanoparticles as structural and electronic modifiers that reinforce conductive pathways without altering the polarity-driven sensitivity trends established above.

#### 3.2.4. Low-Concentration Sensitivity and Interfacial Adsorption Dynamics

**Figure 6:**
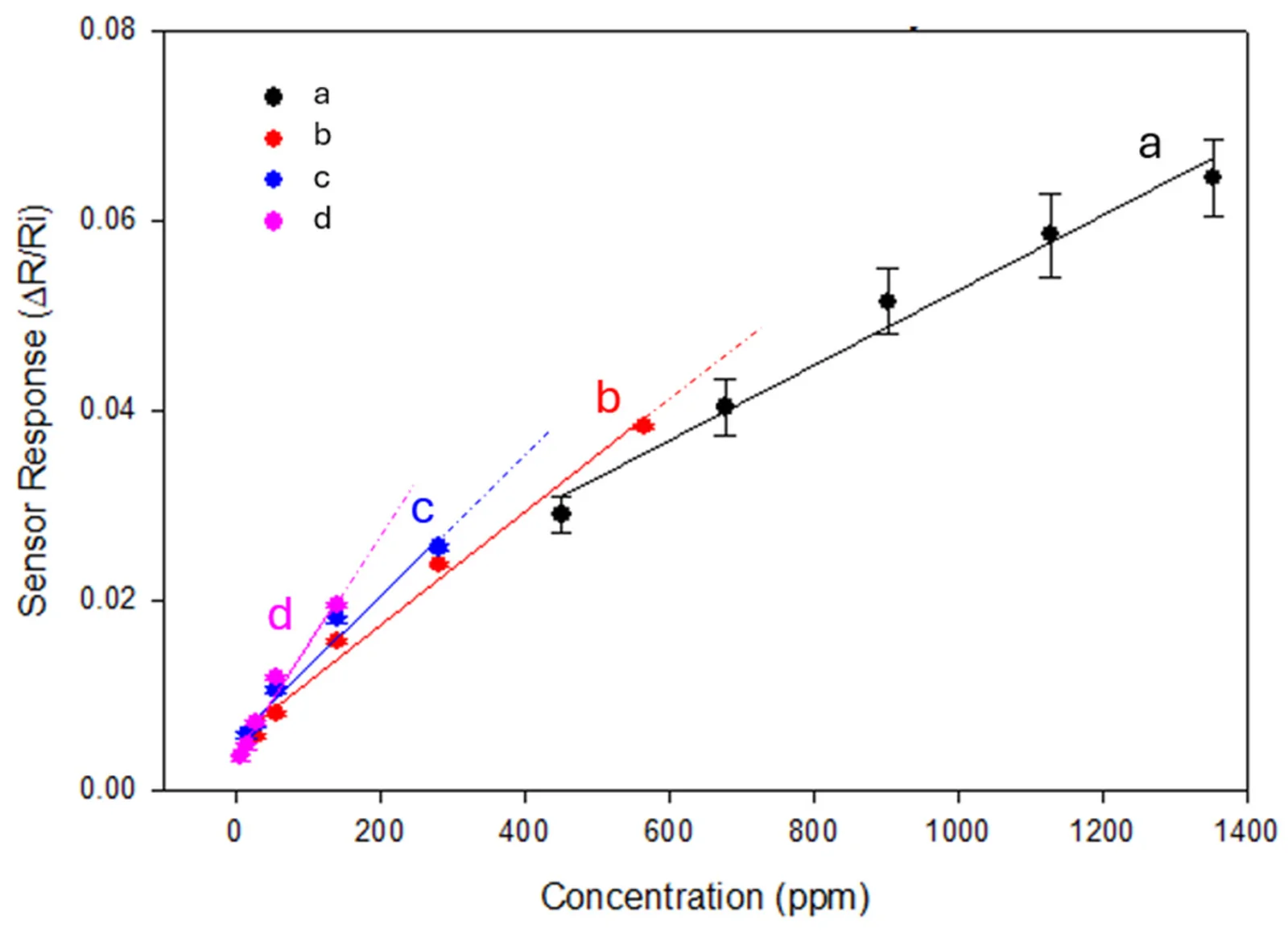
Plots of sensor responses vs. benzene vapor concentration in four different concentration ranges down to as low as ~7 ppm for sensor Au2nm-DT /MUA /GE/HEC. The response sensitivities are: 4.84× 10^−5^ (a), 6.00× 10^−5^ (b), 7.41× 10^−5^ (c), and 1.18× 10^−4^ (d) ppm^−1^. See Figure S6 for details of the custom-built system for controlling different gas/vapor generation, mixing, dilution, and flow conditions (flow rates: 200, 400, 800, and 1600 mL/min). The observation of the enhanced response sensitivity at low VOC concentrations across multiple nanocomposite architectures highlights the importance of interfacial adsorption dynamics toward sub-ppm detection.

A notable feature observed across multiple nanocomposite sensing interfaces is the enhanced chemiresistive sensitivity at low VOC concentrations. Concentration-dependent response measurements reveal that, for several sensors, the slope of ΔR/R_i_ versus concentration increases as VOC levels decrease into the sub-ppm regime (Figure 6). The low concentration was achieved by controlling different gas mixing and dilution under different flow rates (Figure S7). This behavior is consistently observed for both polar and nonpolar VOCs and across different nanocomposite architectures, indicating that it is an intrinsic feature of the sensing interfaces rather than an artifact of a specific material composition. Representative benzene response data illustrate this effect clearly (Figure 6). For GE/HEC-based sensors, the sensitivity below ~50 ppm is significantly higher than that extrapolated from higher concentration ranges, with slopes increasing by up to an order of magnitude depending on the specific nanocomposite formulation. Similar trends are observed for low-vapor pressure VOCs such as xylenes (Figure S8). The result suggests that enhanced low-concentration sensitivity is a general characteristic of the graphene–cellulose-based sensing interfaces examined in this study.

This nonlinear sensitivity behavior is consistent with adsorption processes occurring on heterogeneous nanocomposite surfaces, where high-affinity adsorption sites dominate the response at low surface coverage. At low VOC concentrations, preferential occupation of energetically favorable sites at graphene– and nanoparticle–polymer interfaces leads to disproportionately large perturbations of conductive pathways, resulting in amplified resistance changes. As surface coverage increases at higher concentrations, additional adsorption occurs at lower-affinity sites, producing more gradual changes in resistance and a reduced apparent sensitivity. Langmuir-type adsorption analysis provides further qualitative support for this interpretation. Fits of selected response profiles (Figure S9) indicate faster apparent adsorption kinetics and higher initial uptake rates for sensors exhibiting the strongest sub-ppm sensitivity. While the simplified Langmuir model does not capture all aspects of nanocomposite restructuring or transport limitations, it highlights the important role of interfacial adsorption dynamics in governing chemiresistive response behavior at low analyte concentrations.

From a practical perspective, the enhanced sub-ppm sensitivity observed in these nanocomposite sensors enables detection of VOCs at concentrations relevant to environmental and occupational exposure scenarios. Based on signal-to-noise considerations and regression analysis, several sensing interfaces demonstrate estimated limits of detection in the sub-ppm range for benzene and ethanol, without the need for preconcentration or external amplification. These findings underscore the potential of compositionally programmable nanocomposite sensing interfaces on fibrous substrates as a viable platform for portable, low-cost VOC monitoring and provide important guidance for optimizing sensor arrays toward low-concentration detection (Table S2). Notably, stabilization of the nanocomposite films through repeated exposure and mild annealing further enhances sub-ppm detection by suppressing baseline noise, thereby lowering the practical limit of detection without changing the intrinsic sensitivity trends. This behavior highlights the combined importance of adsorption dynamics and network stability in achieving reliable chemiresistive sensing at low VOC concentrations.

### 3.3. Array-Level Discrimination and Dimensionality Reduction

**Figure 7:**
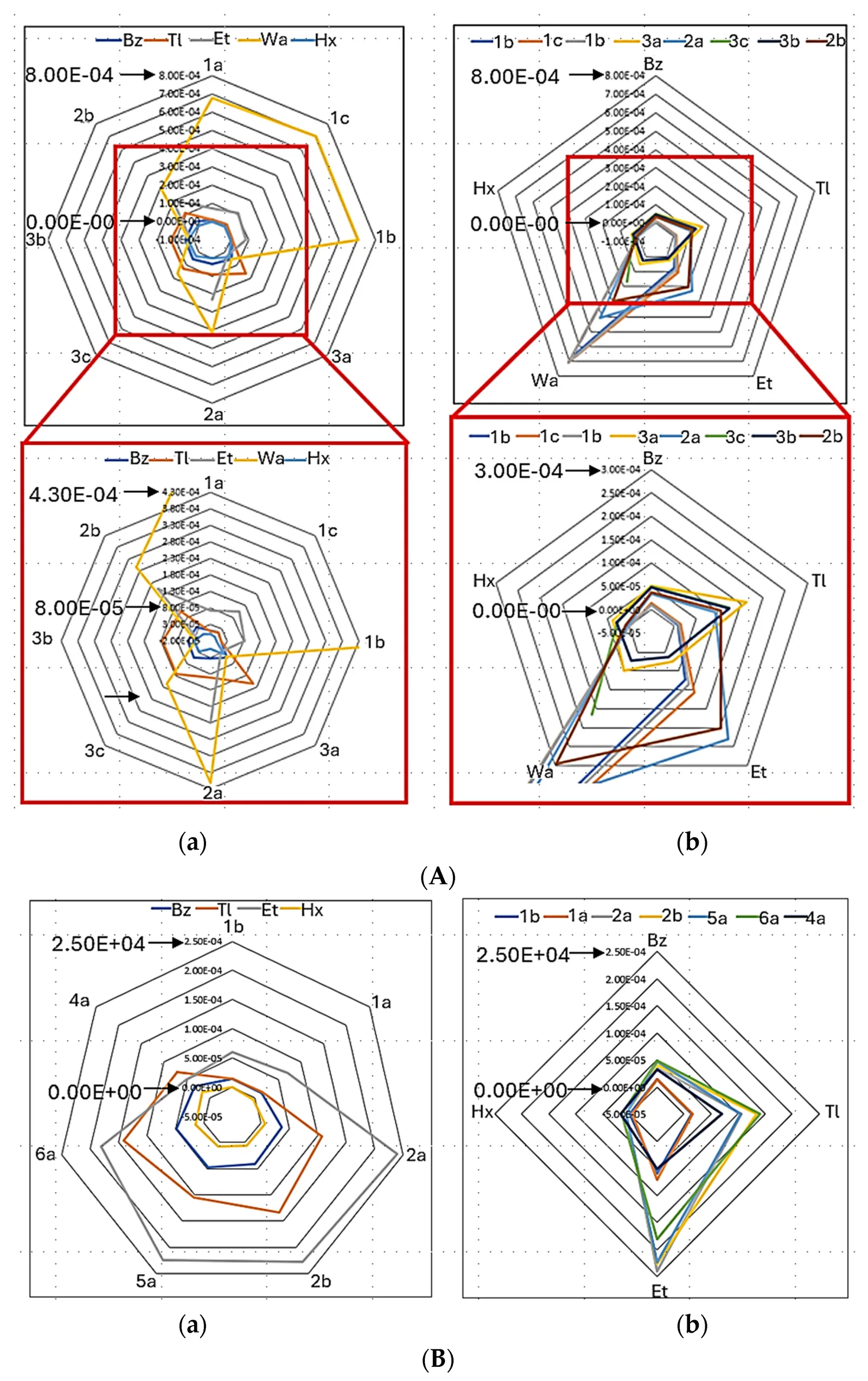
(**A**) Spider charts of the sensor response sensitivity data using array 1 (on IMEs prepared by screen printing and inkjet printing) derived from combinations of nanocomposite components (graphene (GE, carboxymethylcellulose (CMC), 1,9-nonanedithiol (NDT), and Au nanoparticles (Au)) in response to VOCs (hexane (Hx), benzene (Bz), toluene (Tl), ethanol (Et), and water (Wa)). (**B**) Spider charts for data from sensor array 2 in terms of individual VOC response sensitivity (1/ppm) to the individual sensing nanocomposites (a) and the individual sensing nanocomposite’s sensitivity to four individual VOCs (b). For each sub-panel: (i) (**Aa**) presents the sensitivity fingerprints of individual VOCs across the sensors in Array 1. (ii) (**Ab**) presents the complementary sensitivity profile of each Array 1 sensor across the tested VOCs. (iii) (**Ba**) presents the sensitivity fingerprints of individual VOCs across the sensors in Array 2. (iv) (**Bb**) presents the complementary response profile of each Array 2 sensor across the tested VOCs. The radial axes represent sensitivity in ppm^−1^. Note that a positive sensitivity denotes an increase in resistance upon VOC exposure, whereas a negative sensitivity denotes a decrease in resistance with increasing VOC concentration. See Section 2.2 and Table S1 for details of these sensors. The spider charts demonstrate that the tunable nanocomposite array interfaces enable VOC discrimination.

**Figure 8:**
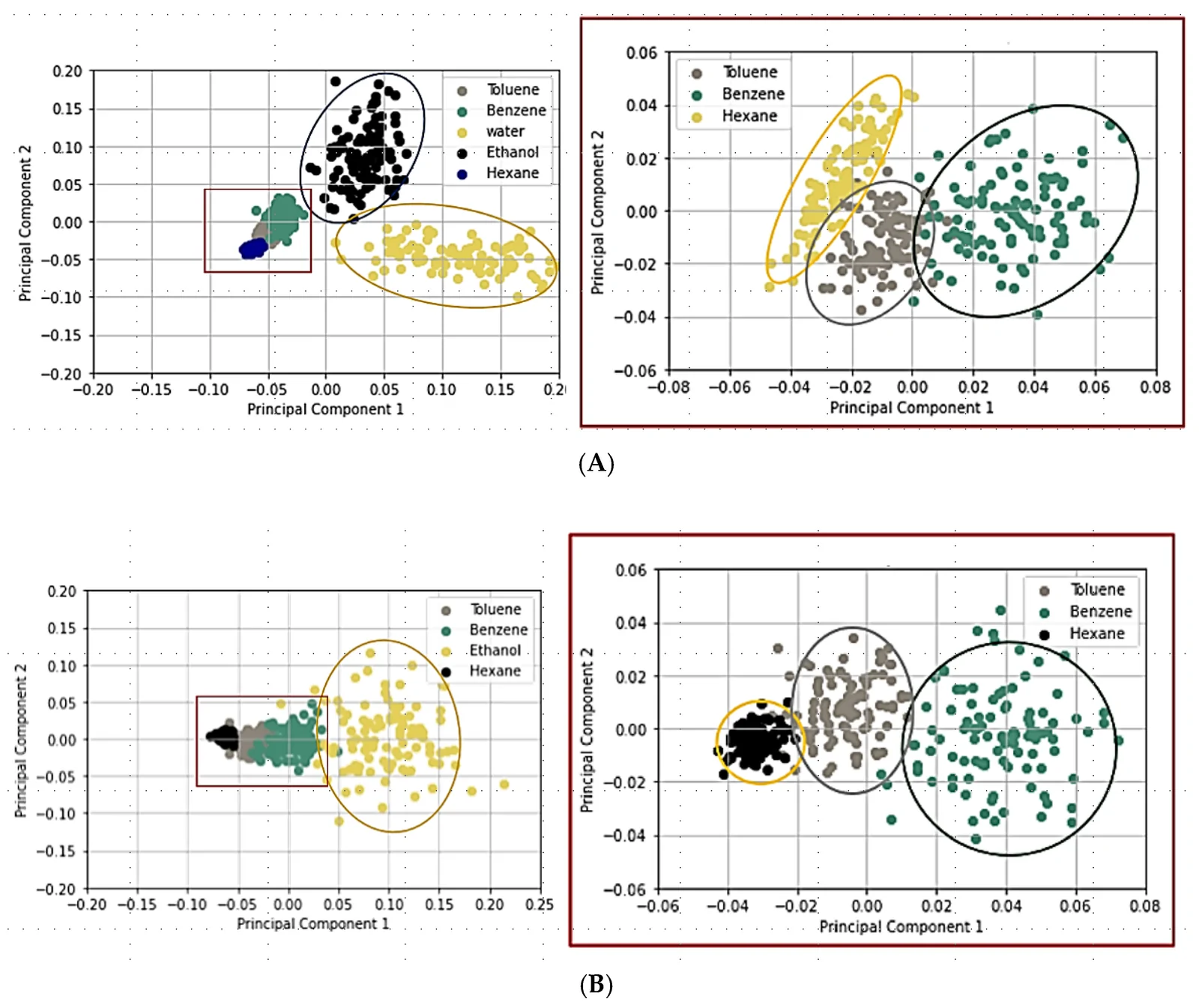
PCA plots of the sensor array responses to different VOCs. (**A**) sensor Array 1. (**B**) sensor Array 2. PC1 (65–80%) captures the overall response magnitude and primary chemical grouping. PC2 (15–25%) captures specific cross-selectivity and fine differentiation between similar vapors. Cumulative variance (85–95%) is high enough to justify omitting further principal components in a 2D plot. These results demonstrate effective separations of VOCs by the array nanocomposite interfaces.

To evaluate the collective discrimination capability of nanocomposite sensor arrays, multivariate analyses were performed using spider-chart visualization and principal component analysis (PCA) (Figure 7 and Figure 8). These methods provide complementary perspectives on how compositional tunability at the individual sensor level translates into array-level selectivity toward chemically diverse VOCs.

Spider charts offer an intuitive representation of relative sensitivity patterns across the array. As shown in Figure 7, sensor arrays composed of nanocomposite interfaces with systematically varied polarity and surface chemistry exhibit distinct sensitivity fingerprints for different VOCs. Hydrophilic and amphiphilic sensing interfaces (GE/CMC and GE/HEC) contribute strongly to responses for polar and hydrogen-bonding vapors, whereas sensors incorporating hydrophobic Au-thiolate assemblies dominate responses to nonpolar aromatic and aliphatic VOCs. The complementary nature of these response patterns enables discrimination among VOC classes based on the collective array response rather than reliance on any single sensor. For some GE/CMC sensors exposed to hexane, the negative slope indicates that adsorption of the hydrophobic vapor produces a net decrease in the measured resistance, likely reflecting a different balance between film swelling, dielectric modulation, and rearrangement of conductive pathways than that observed for the positively responding interfaces. Because the present data does not isolate a single microscopic origin, this behavior is described conservatively as an opposite-direction chemiresistive response rather than assigning a definitive mechanism. The feasibility of modifying or reducing array dimensionality while preserving discrimination capability was also examined. Rational selection of a subset of sensors representing distinct nanocomposite chemistries yields spider-chart patterns that retain the characteristic response contrasts observed in the full array (Figure S10). These results demonstrate that effective VOC discrimination can be achieved with a minimal number of appropriately chosen sensing interfaces, supporting scalable and low-complexity array designs.

PCA further confirms the discrimination capability of the nanocomposite sensor arrays. As shown in Figure 8, sensor responses cluster according to VOC identity with clear separations between polar, hydrophilic, and nonpolar analytes. Comparable clustering behavior is observed for both Array 1 and Array 2, indicating that the design rules established at the individual sensor level translate robustly to array-level performance. Importantly, effective separation is largely retained even when the array is reduced to three or four sensors (Figure S11), although partial overlap between structurally similar VOCs such as benzene and toluene becomes more pronounced.

The results from spider-chart and PCA analyses demonstrate that sensor arrays constructed from compositionally programmable nanocomposite interfaces enable reliable VOC discrimination through chemically complementary response patterns. These results highlight the potential for rationally designed, reduced-complexity chemiresistive sensor arrays for portable and low-cost VOC monitoring applications.

## 4. Conclusions

This work demonstrates a rational and modular strategy for designing chemiresistive sensor arrays based on nanocomposite-structured sensing interfaces on fibrous substrates. By integrating graphene, cellulose derivatives (CMC and HEC), and ligand-functionalized gold nanoparticles, composition-programmable sensing films were constructed in which electrical percolation, surface polarity, and interfacial adsorption characteristics can be systematically tuned. Raman and infrared spectroscopy confirm that graphene incorporation occurs through physical integration rather than chemical modification, preserving polymer integrity while enabling graphene loading to govern charge-transport pathways and chemiresistive sensitivity. Clear design rules emerge linking nanocomposite composition to VOC class sensitivity. Hydrophilic GE/CMC and amphiphilic GE/HEC interfaces preferentially enhance responses to polar and hydrogen-bonding VOCs, whereas hydrophobic Au-thiolate assemblies exhibit stronger responses to nonpolar aromatic and aliphatic vapors. Incorporation of ligand-functionalized gold nanoparticles introduces an additional tunability dimension, enabling modulation of sensitivity, linearity, and response stability without disrupting the underlying graphene–cellulose architecture. Across multiple nanocomposite formulations, enhanced sensitivity in the sub-ppm concentration regime is observed, highlighting the importance of interfacial adsorption dynamics and network stability for practical VOC detection.

At the array level, complementary nanocomposite interfaces generate distinct sensitivity fingerprints that enable reliable discrimination among chemically diverse VOCs, as demonstrated by spider-chart analysis and principal component analysis. Reduced-sensor configurations retain the principal VOC-class separation trends, but partial overlap becomes more pronounced for structurally similar aromatic VOCs, particularly benzene and toluene. These results establish compositionally programmable nanocomposite interfaces as a versatile platform for chemiresistive VOC sensing and provide actionable design guidelines for developing portable, low-cost sensor arrays for environmental and occupational exposure monitoring. Systematic investigations under controlled relative humidity and in the presence of common interfering gases, including formaldehyde, acetone, and CO, constitute important future studies toward practical environmental deployment of the sensor arrays.

## Data Availability

The original contributions presented in this study are included in the article/Supplementary Material. Further inquiries can be directed to the corresponding author.

## Supplementary Materials

The following supporting information can be downloaded at: https://www.mdpi.com/article/10.3390/s26175369/s1.
