# Supplementary material for: Nanocomposite-Structured Sensing Interfaces on Fibrous Substrates for Chemiresistive Detection of VOCs
Source: Sensors (Basel). 2026 Aug 25;26(17):5369. doi: 10.3390/s26175369 (PMC13568310; doi:10.3390/s26175369)
Supplement: Supplementary file 1 [file sensors-26-05369-s001.zip › sensors-4471967-supplementary.pdf]

Supporting Information

Nanocomposite-Structured Sensing Interfaces on Fibrous Substrates for Chemiresistive Detection of VOCs

Lidia Gebre 1, Guojun Shang 1, Zeqi Li 1, Dong Dinh 2, Seyed Danial Mousavi 1, Madelyn Lee 1, Ielyzaveta Antonova 1, Jin Luo 1, Susan Lu 2, Cate Wisdom 3,4, Emily Long 3,4, Zakiya Skeete 1, Tony Yuan 3,5,* and Chuan-Jian Zhong 1,*

[1] Department of Chemistry, State University of New York at Binghamton, Binghamton, NY 13902, USA [2] Department of Systems Science and Industrial Engineering, State University of New York at Binghamton, Binghamton, NY 13902, USA [3] 4D Bio3 Center for Biotechnology, Uniformed Services University , Bethesda, MD 20814, USA [4] The Geneva Foundation, Tacoma, WA 98402, USA [5] Department of Radiology and Bioengineering, Uniformed Services University, Bethesda, MD 20814, USA

Table S1: General characteristics of sensor Array-1 (8 sensors) and Array-2 (7 sensors)

Sensor #	Sensor Group	Sensor type
Array-1		
S-1	1a	GE/CMC
S-3	1b	GE/CMC
S-2	1c	GE/CMC
S-5	2a	GE/HEC
S-8	2b	GE/HEC
S-4	3a	Au_{2nm}-NDT
S-7	3b	Au_{2nm}-NDT
S-6	3c	Au_{2nm}-NDT
Array-2		
S-2	1a	GE/CMC
S-1	1b	GE/CMC
S-3	2a	GE/HEC
S-4	2b	GE/HEC
S-5	-	-
S-8	4a	Au_{2nm}-DT /GE/CMC
S-6	5a	Au_{2nm}-DT /GE/HEC
S-7	6a	Au_{2nm}-DT /MUA /GE/HEC

Notes: (1) mixing ratio of GE:CMC or GE:HEC: 1:1. The structures are shown below:

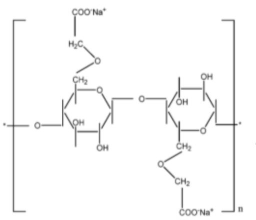
Sodium Carboxymethylcellulose (CMC)

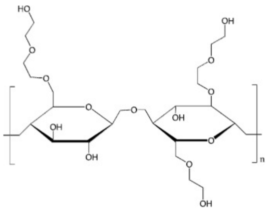
Hydroxyethylcellulose (HEC)

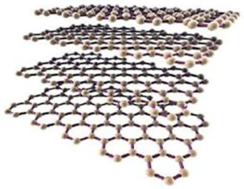
Graphene Nanoplatelets (GE)

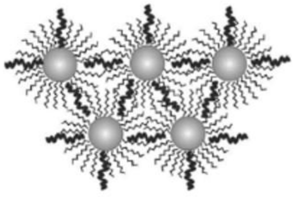
Au nanoparticles capped with nonanedithiol (NDT)

(2) Descriptions of the Arrays: The order of the sensors was based on the film type, not the sensor position number in the array socket.

In the 8-sensor array socket (Array-1), the sensors are arranged linearly in a row from 1 to 8 and designated as S-1 through S-8. These sensors are classified into three distinct categories based on their chemical composition and structural type:

i. Group 1 (GE/CMC): Comprises sensors S-1, S-3, and S-2, subdivided into groups 1a, 1b, and 1c respectively.
ii. Group 2 (GE/HEC): Comprises sensors S-5 and S-8, subdivided into groups 2a and 2b respectively.
iii. Group 3 (Au2nm-NDT): Comprises sensors S-4, S-7, and S-6, subdivided into groups 3a, 3b, and 3c respectively.

In the second array configuration (Array-2), the socket accommodates 7 functional sensors labeled from S-1 to S-8 (with S-5 left unassigned). These sensors are classified into five distinct categories based on their chemical composition and structural type:

i. Group 1 (GE/CMC): Comprises sensors S-2 and S-1, subdivided into groups 1a and 1b respectively.
ii. Group 2 (GE/HEC): Comprises sensors S-3 and S-4, subdivided into groups 2a and 2b respectively.
iii. Unassigned (S-5): The position for Sensor S-5 contains no sensor.
iv. Group 4 (Au2nm-DT / GE/CMC): Contains sensor S-8, designated as group 4a.
v. Group 5 (Au2nm-DT / GE/HEC): Contains sensor S-6, designated as group 5a.
vi. Group 6 (Au2nm-DT / MUA / GE/HEC): Contains sensor S-7, designated as group 6a.

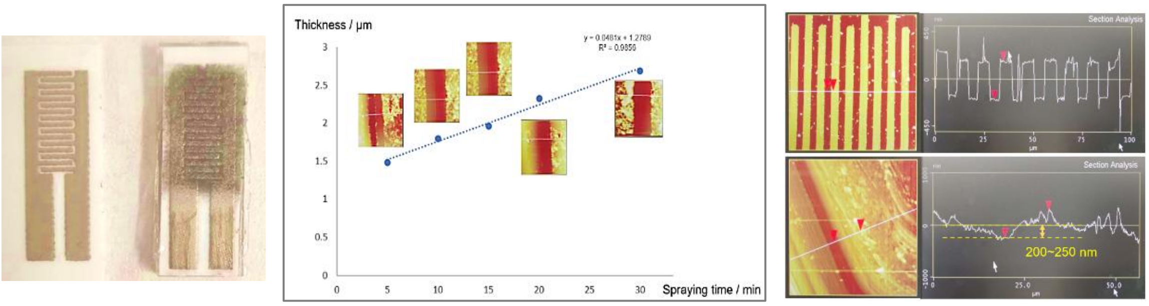

(A) (B) (C)

Figure S1. (A) Photo showing a representative screen-printed IME before (left) and after (right) spraying nanocomposite film; (B) plot of film thickness vs. spraying time (insets: AFM images of the film with a scratch line on a mica surface; (C) AFM images of microelectrode and thin film thickness analysis (on mica).

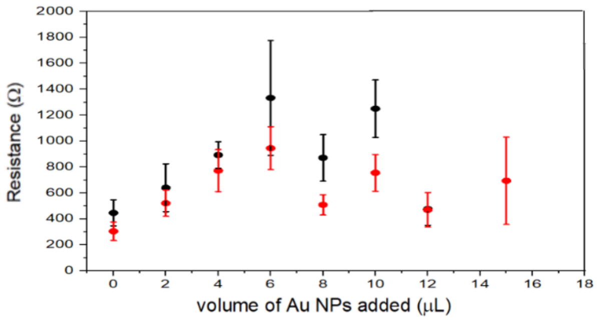

Figure S2. Plot of electrical resistance of the nanocomposite films vs. the amount of Au_{2nm}-MUA component. The graphene - CMC solution has a 1:10 GE/CMC mass ratio and the Au NPs (Au_{2nm}-MUA has a concentration of 0.005 g/mL.

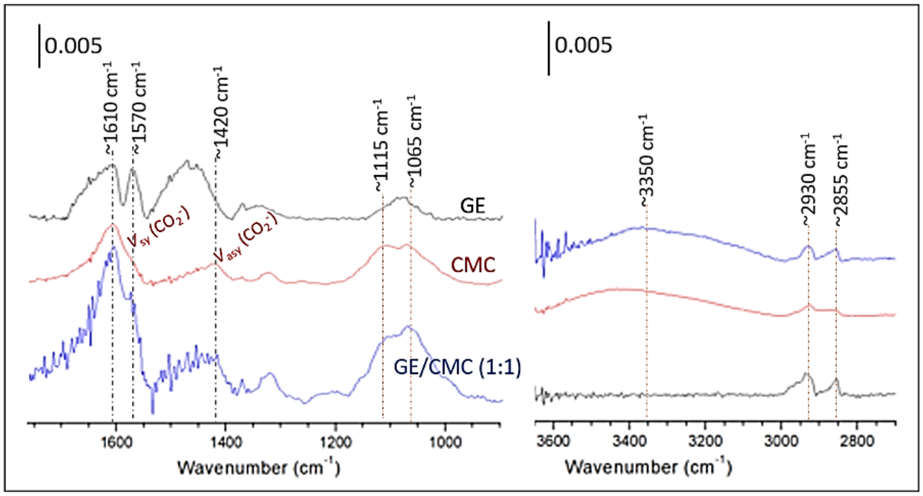

Figure S3. Infrared reflection–absorption spectra (IRRAS) of pure CMC, GE, and GE/CMC nanocomposite films deposited on gold-coated fibrous substrates.

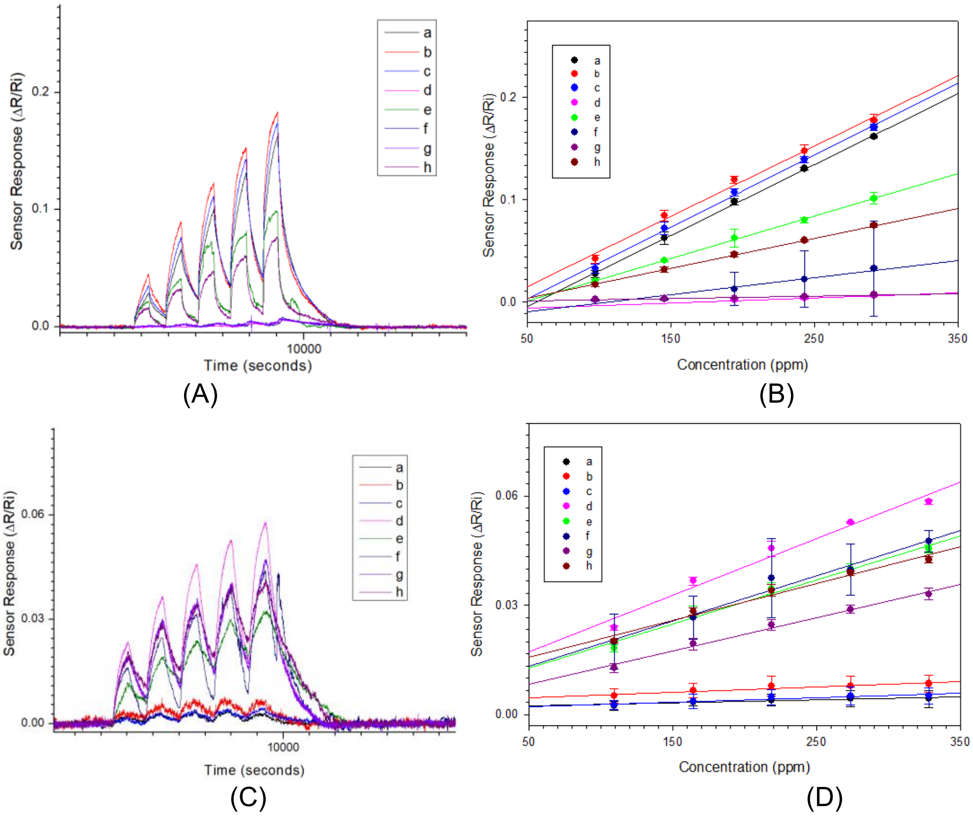

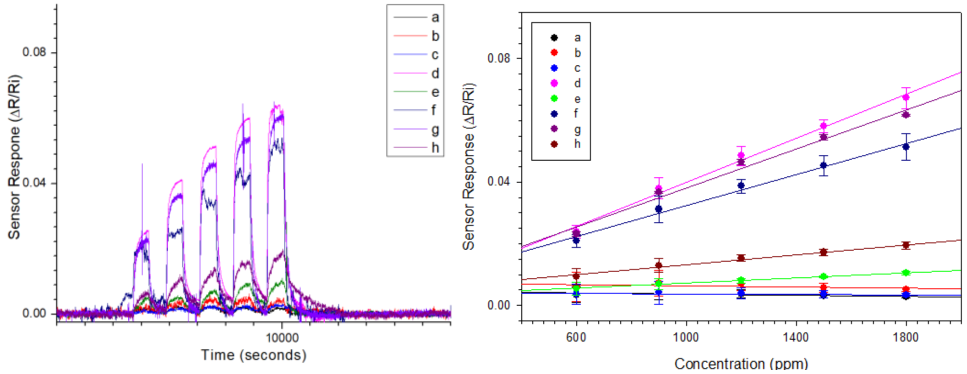

(E) (F)

Figure S4. Sensor responses and sensitivities in response to VOCs: (A-B) water vapor. (A) sensor (S) response profile of array 1: sensor 1; b: sensor 2; c: sensor 3; d: sensor 4; e: sensor 5; f: sensor 6 g: sensor 7; h: sensor 8. (B) sensitivity plot of array 1 against water vapors (a: 6.79 x 10^{-4} 1/ppm; b: 7.07 x 10^{-4} 1/ppm; c: 7.04 x 10^{-4} 1/ppm; d: 4.90 x 10^{-5} 1/ppm; e: 4.12 x 10^{-4} 1/ppm; f: 1.66 x 10^{-4} 1/ppm; g: 2.33 x 10^{-5} 1/ppm; h: 2.95 x 10^{-4} 1/ppm). (C-D) toluene vapor. (C) sensor response profile of array 1: sensor 1; b: sensor 2; c: sensor 3; d: sensor 4; e: sensor 5; f: sensor 6 g: sensor 7; h: sensor 8. (D) sensitivity plot of array 1 against toluene vapors (a: 8.47 x 10^{-6} 1/ppm; b: 1.46 x 10^{-5} 1/ppm; c: 1.19 x 10^{-5} 1/ppm; d: 1.64 x 10^{-4} 1/ppm; e: 9.28 x 10^{-5} 1/ppm; f: 1.25 x 10^{-4} 1/ppm; g: 1.24 x 10^{-4} 1/ppm; h: 1.06 x 10^{-4} 1/ppm). (E-F) hexane vapor. (E) sensor response profile of array 1: sensor 1; b: sensor 2; c: sensor 3; d: sensor 4; e: sensor 5; f: sensor 6 g: sensor 7; h: sensor 8. (F) sensitivity plot of array 1 against hexane vapors (a: -8.38 x 10^{-7} 1/ppm; b: -9.59 x 10^{-7} 1/ppm; c: -3.39 x 10^{-7} 1/ppm; d: 3.68 x 10^{-5} 1/ppm; e: 3.98 x 10^{-6} 1/ppm; f: 2.66 x 10^{-5} 1/ppm; g: 2.93 x 10^{-5} 1/ppm; h: 7.49 x 10^{-6} 1/ppm)

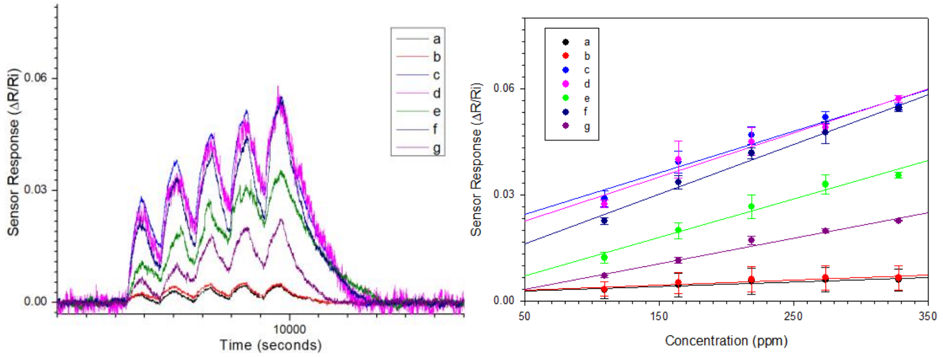

(A) (B)

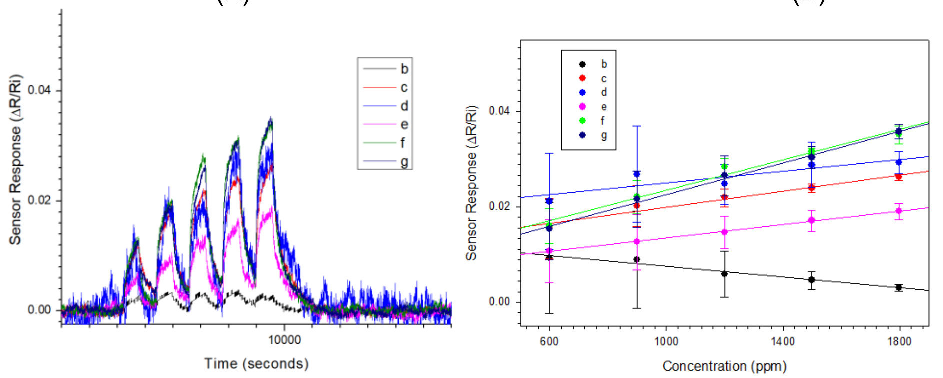

(C) (D)

Figure S5: Sensor responses and sensitivities in response to VOCs. (A-B) toluene vapor. (A) Sensor response profile of array 2: sensor 1; b: sensor 2; c: sensor 3; d: sensor 4; e: sensor 6; f: sensor 7; g: sensor 8. (B) sensitivity plot of array 2 against toluene vapors (a: 1.41 x 10^{-5} 1/ppm; b: 1.60 x 10^{-5} 1/ppm; c: 1.07 x 10^{-4} 1/ppm; d: 1.34 x 10^{-4} 1/ppm; e: 1.04 x 10^{-4} 1/ppm; f: 1.41 x 10^{-4} 1/ppm; g: 7.05 x 10^{-5} 1/ppm). (C-D)

hexane vapor. (C) Sensor response profile of array 2: b: sensor 2; c: sensor 3; d: sensor 4; e: sensor 6; f: sensor 7; g: sensor 8. (D) sensitivity plot of array 2 against hexane vapors (b: -5.22×10^{-6} 1/ppm; c: 7.45×10^{-6} 1/ppm; d: 6.19×10^{-6} 1/ppm; e: 7.05×10^{-6} 1/ppm; f: 1.49×10^{-5} 1/ppm; g: 1.65×10^{-5} 1/ppm).

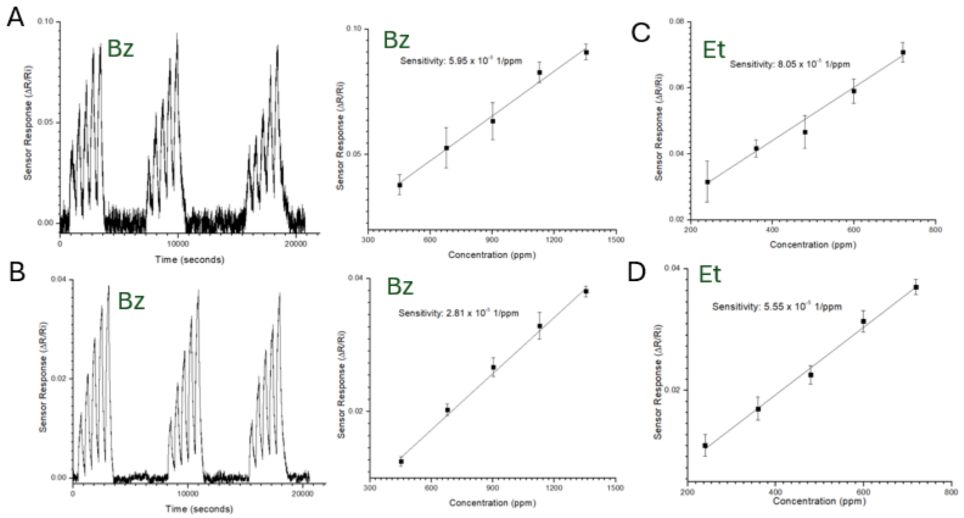

Figure S6. Response profiles and response sensitivities of Au-DT/GE/CMC sensor in response to benzene (Bz) (A-B) and ethanol (Et) (C-D) before and after room temperature annealing from VOC exposures. Three replicas in each case, the benzene concentrations are indicated in the linear plots. (array 2). Repeated benzene measurements performed after approximately **three months** of ambient storage exhibited stable baseline resistance and reproducible sensing responses, demonstrating good storage stability and reproducibility of the nanocomposite sensing interfaces over extended periods.

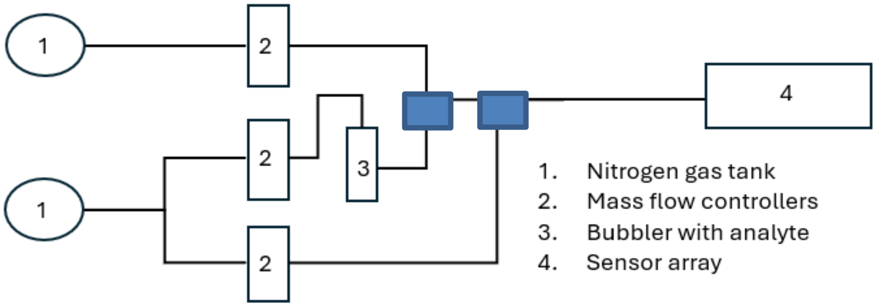

Figure S7. Custom-built system for controlling different gas/vapor generation, mixing, dilution, and flow conditions, including a single dilution for high vapor concentrations (>200 ppm), and two-consecutive dilutions for low vapor concentrations (as low as sub ppm). Blue boxes: dilution valves.

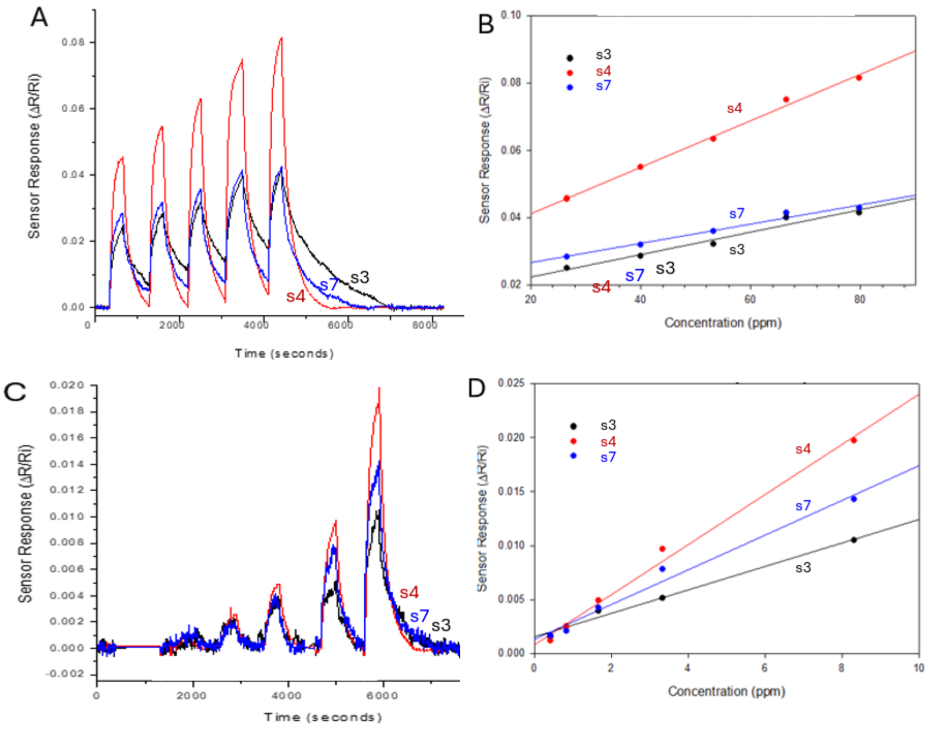

Figure S8: Representative response profiles and response sensitivities of high (A-B) and low (C-D) concentration of o-xylene. Response sensitivities: (B) 3.35 x 10^{-4} (s3), 6.91 x 10^{-4} (s4), and 2.88 x 10^{-4} ppm^{-1} (s7); (D) 1.09 x 10^{-3} (s3), 2.32 x 10^{-3} (s4), and 1.61 x 10^{-3} ppm^{-1} (s7). Note that the sensing interfaces retained their characteristic response behavior with no observable deterioration in baseline stability or sensing trends after approximately 15 months after the original Array 1 and Array 2 measurements using the same sensing platform stored under ambient laboratory conditions.

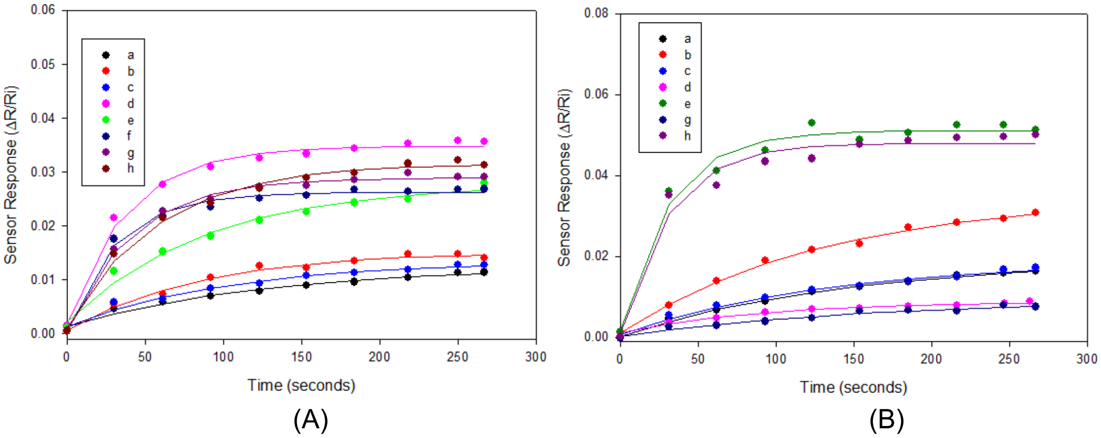

Figure S9. Langmuir adsorption model fitting to the sensor response vs. time curves and rate constant calculation from the first vapor concentration response for (A) benzene and (B) ethanol vapors (array 1). Rate constant (1/s) calculation for each sensor, a: S-1; b: S-2; c: S-3; d: S-4; e: S-5; f: S-6 g: S-7; h: S-8. (A): a: 0.0079; b: 0.0118; c: 0.0096; d: 0.0265; e: 0.0111; f: 0.0318; g: 0.0239; h: 0.0171. (B): a: 0.0068; b: 0.0076; c: 0.0073; d: 0.0106; e: 0.0323; f: (no response); g: 0.0062; h: 0.0320.

Table S2. Summary of response rate constants and response times for (A) benzene and (B) ethanol vapors (array 1) based on the data shown in Figure S9.

Sensor	Benzene Rate Constant (k, s^{-1})	Benzene t_{90} (s)	Ethanol Rate Constant (k, s^{-1})	Ethanol t_{90} (s)
S-1 (a)	0.0079	291	0.0068	338
S-2 (b)	0.0118	195	0.0076	303
S-3 (c)	0.0096	239	0.0073	315
S-4 (d)	0.0265	86	0.0106	217
S-5 (e)	0.0111	207	0.0323	71
S-6 (f)	0.0318	72	-	-
S-7 (g)	0.0239	96	0.0062	371
S-8 (h)	0.0171	134	0.0320	72

Table S3. Visually estimated recovery time (the time required for the signal to decay to 10% of its maximum peak value (t10))

Sensor	Benzene Recovery Time (s)	Ethanol Recovery Time (s)
**S-1 (a)**	~300 s *(low signal)*	~400 s *(low signal)*
**S-2 (b)**	~350 s	~350 s
**S-3 (c)**	~300 s	~400 s
**S-4 (d)**	~250 s	~300 s
**S-5 (e)**	~400 s	~150 s
**S-6 (f)**	~150 s	*No Response*
**S-7 (g)**	~300 s	~400 s
**S-8 (h)**	~350 s	~150 s

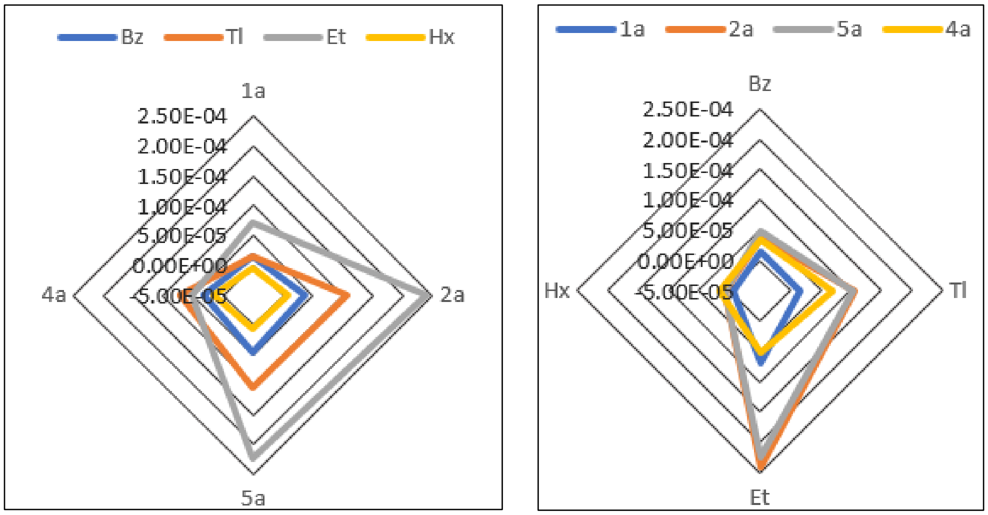

(A)

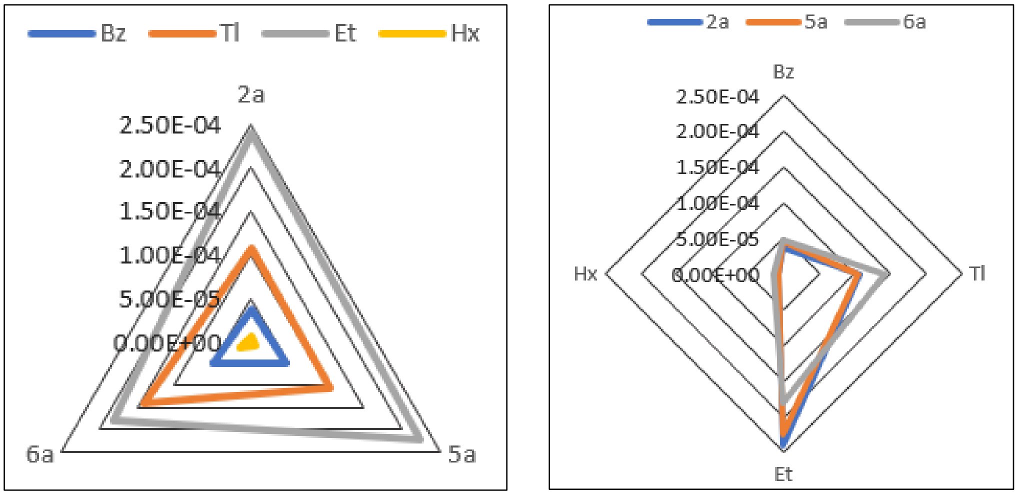

(B)

Figure S10. Spider charts with reduced number of sensors in the array (array-2). (A) 4-sensors: GE/CMC (1a), GE/HEC (2a), Au2nm-DT w/ 1:1 GE/HEC (5a), Au2nm-DT w/ 1:1 GE/CMC (4a); (B) 3 sensors: GE/HEC (2a), Au2nm-DT w/ 1:1 GE/HEC (5a), Au2nm-MUA w/ 1:1 GE/HEC (6a).

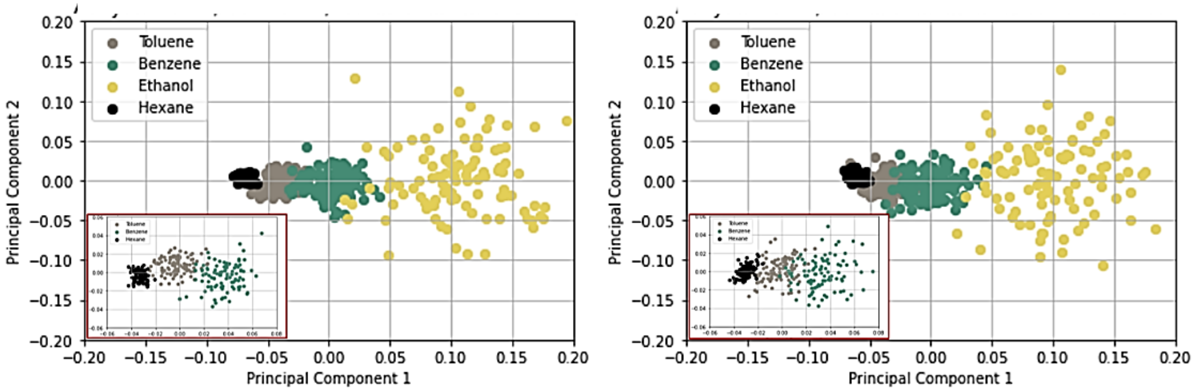

(A) (B)

Figure S11. PCA plots of different sensor arrays (from Array 2) responses to different VOCs. (A) 4-sensor array: GE/CMC (2), GE/HEC (3), Au_{2nm}-DT w/ 1:1 GE/HEC (6), and Au_{2nm}-DT w/ 1:1 GE/CMC (8); (B) 3 sensor array: GE/HEC (3), Au_{2nm}-DT w/ 1:1 GE/HEC (6), and Au_{2nm}-MUA w/ 1:1 GE/HEC (7). The insets are magnified views of the data for the toluene, benzene and hexane.

Table S4. Summary of VOC sensing performance of representative sensing interfaces, including sensitivity, estimated limits of detection, and apparent adsorption kinetics for selected VOCs

Sensors	VOC	Sensitivity (ppm^{-1})	LOD (ppm)	Apparent Rate Constant k (s^{-1})	Key Feature / Note
GE/HEC (Array 1)	Ethanol	2.30×10^{-4}	.456	0.0323	amphiphilic interface; high ethanol sensitivity
Au–NDT (Array 1)	Benzene	5.10×10^{-5}	2.06	0.0265	hydrophobic interface; strong aromatic response
Au–DT/GE/HEC (Array 2)	Benzene	4.67×10^{-5}	2.25	–	ternary composite; improved stability

Sensors	VOC	Sensitivity (ppm^{-1})	LOD (ppm)	Apparent Rate Constant k (s^{-1})	Key Feature / Note
Au–DT/MUA/GE/HEC (Array 2)	Ethanol	1.81×10^{-4}	.580	–	ligand-functionalized tunability

Table S5. Comparison of representative recent chemiresistive VOC sensors based on paper/fibrous substrates, graphene/carbon nanomaterials, and nanoparticle-containing sensing interfaces with representative nanocomposite sensors developed in this work.

Sensing material / interface	Substrate / platform	Target VOC(s)	Sensitivity / response	LOD / lowest reported concentration	Response / recovery time	Stability / humidity	Ref.
Graphene–PEDOT:PSS/WS_2 nanotubes	Conductive paper	Butanol, toluene, methanol, acetone	Linear chemiresistive responses; numerical sensitivity not reported	44.92 ppm (butanol)	205 / 20 s (butanol)	NR	1
Carbon black/exfoliated graphene	Filter paper	Methanol, ethanol, toluene, dichloromethane, acetone, petroleum vapor	Detectable responses to multiple VOCs	NR	76 / 115 s (methanol); 225 / 245 s (ethanol); 447 / 812 s (toluene); other VOCs also reported	Operable over RH 33–95%	2
Au NPs capped with DT/DD; surface-mediated nanoparticle assemblies	PAN/PET fibrous membrane	Methanol, ethanol, 1-propanol, 1-butanol, 1-pentanol	Response magnitude and direction vary systematically with alcohol chain length and ligand chemistry	NR	NR	NR	3
**Bacterial cellulose/PEG aerogel + graphene + TiO_2 or ZnO nanoparticles**	3D fibrous bacterial-cellulose aerogel	Acetone, formaldehyde, ethanol	High VOC sensitivity/selectivity; composition-dependent response	**1.43 ppm acetone**	**~20 s response to 1 ppm acetone**	NR	4
**LIG–CoPc/MWCNT nanocomposite**	Flexible laser-induced graphene/PI platform	Methanol; selectivity tested against ethanol, isopropanol, acetone	**0.589 Ω ppm^{-1}**	**0.165 ppm methanol**	**5 s (t45); 164 s (t85 response); 108 s (t85 recovery)** at 500 ppm	**<4.25% degradation over 30 days**	5
Au-, Pd-, and Pt-decorated graphene	Alumina	Ethanol, xylene and other VOCs	Au–graphene: 0.501% (ethanol), 1.982% (xylene)	~0.15 ppm for Au–graphene	495 / 489 s (ethanol); 252 / 682 s (xylene)	NR	6
**GE/HEC, S-5, Array 1**	Printed paper IME	Ethanol	**2.30×10^{-4} ppm^{-1}**	**0.456 ppm**	**71 / ~150 s**	Response behavior retained in extended-storage testing	**7**
**GE/HEC, S-8, Array 1**	Printed paper IME	Ethanol	**2.01×10^{-4} ppm^{-1}**	—	**72 / ~150 s**	Response behavior retained in extended-storage testing	**7**
**Au_2nm–NDT, S-4, Array 1**	Printed paper IME	Benzene	**5.10×10^{-5} ppm^{-1}**	**2.06 ppm**	**86 / ~250 s**	Stable response after ambient storage testing	**7**

Sensing material / interface	Substrate / platform	Target VOC(s)	Sensitivity / response	LOD / lowest reported concentration	Response / recovery time	Stability / humidity	Ref.
**Au_2nm–NDT, S-6, Array 1**	Printed paper IME	Benzene	**5.06×10^{-5} ppm^{-1}**	—	**72 / ~150 s**	Stable response after ambient storage testing	**7**
**Au_2nm–DT/GE/HEC, Array 2**	Printed paper IME	Benzene	**4.67×10^{-5} ppm^{-1}**	**2.25 ppm**	NR	Improved cycle-to-cycle stability relative to unmodified GE/HEC	**7**
**Au_2nm–DT/MUA/GE/HEC, Array 2**	Printed paper IME	Ethanol	**1.81×10^{-4} ppm^{-1}**	**0.580 ppm**	NR	Ligand-functionalized ternary interface; improved response stability	**7**

References for Table S5

1. Huang, S.-J.; Immanuel, P. N.; Yen, Y.-K.; Yen, C.-L.; Tseng, C.-E.; Lin, G.-T.; Lin, C.-K.; Huang, Z.-X. Tungsten Disulfide Nanotube-Modified Conductive Paper-Based Chemiresistive Sensor for the Application in Volatile Organic Compounds’ Detection. *Sensors* 2021, *21* (18), 6121.
2. Liu, H.; Zheng, H.; Xiang, H.; Wang, W.; Wu, H.; Li, Z.; Zhuang, J.; Zhou, H. Paper-Based Wearable Sensors for Humidity and VOC Detection. *ACS Sustainable Chemistry & Engineering* 2021, *9* (50), 16937–16945.
3. Yan, S.; Dinh, D. ; Shang, G. ; Wang, S. ; Zhao, W.; Liu, X.; Robinson, R.; Lombardi III, J.; He, N.; Lu, S.; Poliks, M.; Hsiao, B.; Gitsov, I.; Zhong, C. J., Nano-Filamented Textile Sensor Platform with High Structure Sensitivity, ACS Applied Materials Interfaces, 2022, 14, 15391–15400
4. Wei, W.; Song, S.; Meng, C.; Li, R.; Feng, Y.; Chen, X.; Chang, J.; Fei, B.; Yang, W.; Li, J. Bacterial Cellulose/Polyethylene Glycol Composite Aerogel with Incorporated Graphene and Metal Oxides for VOCs Detection. *Chemical Engineering Journal* 2024, *499*, 156510.
5. Lu, T.; Adiraju, A.; Lyu, A.; Cui, Z.; Shi, G.; Al-Hamry, A.; Pašti, I. A.; Kanoun, O. Flexible Gas Sensor Based on Laser-Induced Graphene and Cobalt Phthalocyanine-MWCNTs Composite for Methanol Detection at Room Temperature. *Emergent Materials* 2025, *8* (4), 2815–2829.
6. Behi, S.; Thamri, A.; Casanova-Chafer, J.; Karageorgos Perez, N.; Eduard Llobet, Adnane Abdelghani, Detection of VOCs Using Metal Nanoparticle-Decorated Graphene. *Chemosensors* 2026, *14* (5), 111.
7. This work.
